# Enhanced detection of cortical atrophy in Alzheimer's disease using structural MRI with anatomically constrained longitudinal registration

**DOI:** 10.1002/hbm.25455

**Published:** 2021-05-14

**Authors:** Emily Iannopollo, Kara Garcia, Alzheimer' Disease Neuroimaging Initiative

**Affiliations:** ^1^ Department of Radiology and Imaging Sciences Indiana University School of Medicine Evansville Indiana USA

**Keywords:** Alzheimer disease, cerebral cortex, longitudinal registration, magnetic resonance imaging, mild cognitive impairment, neurodegeneration, neuroimaging

## Abstract

Cortical atrophy is a defining feature of Alzheimer's disease (AD), often detectable before symptoms arise. In surface‐based analyses, studies have commonly focused on cortical thinning while overlooking the impact of loss in surface area. To capture the impact of both cortical thinning and surface area loss, we used anatomically constrained Multimodal Surface Matching (aMSM), a recently developed tool for mapping change in surface area. We examined cortical atrophy over 2 years in cognitively normal subjects and subjects with diagnoses of stable mild cognitive impairment, mild cognitive impairment that converted to AD, and AD. Magnetic resonance imaging scans were segmented and registered to a common atlas using previously described techniques (FreeSurfer and ciftify), then longitudinally registered with aMSM. Changes in cortical thickness, surface area, and volume were mapped within each diagnostic group, and groups were compared statistically. Changes in thickness and surface area detected atrophy at similar levels of significance, though regions of atrophy somewhat differed. Furthermore, we found that surface area maps offered greater consistency across scanners (3.0 vs. 1.5 T). Comparisons to the FreeSurfer longitudinal pipeline and parcellation‐based (region‐of‐interest) analysis suggest that aMSM may allow more robust detection of atrophy, particularly in earlier disease stages and using smaller sample sizes.

## Graphical abstract

To capture the impact of both cortical thinning and surface area loss in Alzheimer's disease, we used anatomically constrained Multimodal Surface Matching (aMSM), a recently developed tool for mapping change in surface area. Changes in thickness and surface area detected atrophy at similar levels of significance, though regions of atrophy somewhat differed. Comparisons to the FreeSurfer longitudinal pipeline and parcellation‐based (region‐of‐interest) analysis suggest that aMSM may allow more robust detection of atrophy, particularly in earlier disease stages and using smaller sample sizes.

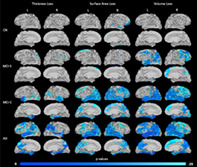

## 1 INTRODUCTION

Alzheimer's disease (AD) is a debilitating neurodegenerative condition affecting 10% of Americans age 65 or older (Alzheimer's Association, 2020). The disease often progresses from early clinical symptoms, such as lapses in memory, apathy, and depression, to more severe symptoms including behavior changes, confusion, and difficulty with basic functions such as speaking, swallowing, and walking (Alzheimer's Association, 2020). The onset of AD is insidious, with years between initial biochemical changes and the onset of clinically apparent symptoms (Brookmeyer, Abdalla, Kawas, & Corrada, 2018). This pattern of slow progression offers a window for early detection and has raised the need for noninvasive methods of tracking AD‐related changes.

Magnetic resonance imaging (MRI), which can be used to monitor the cortical changes that accompany AD, has become a useful tool in addressing this need (Chandra, Dervenoulas, Politis, & ADNI, 2019; Femminella et al., 2018). Three‐dimensional reconstructions derived from MRI allow for analysis of cortical anatomy, and surface‐based methods of analyzing these reconstructions have proven beneficial (Clarkson et al., 2011; Glasser et al., 2013; Robinson et al., 2018). Within the surface‐based methodologies, previous studies have often examined changes in cortical thickness as a marker of atrophy in AD (Belathur Suresh, Fischl, Salat, & ADNI, 2018; Clarkson et al., 2011; Cuingnet et al., 2011; Mattsson et al., 2018; Ossenkoppele et al., 2019). However, in this study we propose that vertex‐wise mapping of cortical surface area changes, in addition to cortical thickness changes, could offer an improved method of tracking cortical atrophy over time.

A recently developed tool, anatomically constrained Multimodal Surface Matching (aMSM), has been shown to enable more accurate tracking of physical cortical changes over time, in a single subject (Robinson et al., 2018). aMSM constrains local deformations of the anatomical surface to realistic behavior, based on the mechanical properties of brain tissue, producing detailed, vertex‐wise maps of change in surface area. This methodology has previously been used to track cortical growth (Garcia et al., 2018), but it has not yet been leveraged to measure patterns of cortical atrophy. As loss of cortical volume is dependent on both changes in thickness and changes in surface area, detailed maps of surface area loss, in combination with maps of cortical thinning, offer the ability to better characterize degenerative changes occurring across the cortex in AD.

In this study, we used MRI data from the Alzheimer's Disease Neuroimaging Initiative (ADNI) to test the utility of aMSM in accurately detecting patterns of cortical atrophy. Using aMSM, we mapped changes in both thickness and surface area over 2 years in individual subjects with diagnoses of cognitively normal (CN), stable mild cognitive impairment (MCI‐S), mild cognitive impairment which converted to AD (MCI‐C), and AD. First, each diagnostic group was evaluated to identify patterns of atrophy in terms of thickness, surface area, and volume loss. Then, comparisons between groups were used to identify diagnostically relevant differences in atrophy. To compare our approach to existing methodologies, we repeated our analysis using the FreeSurfer longitudinal pipeline (Reuter, Schmansky, Rosas, & Fischl, 2012) and region‐of‐interest (ROI) analysis. Next, to examine the precision of aMSM‐generated measures of atrophy across imaging methods, we compared atrophy maps from a subset of subjects who were scanned using two different acquisition protocols (3.0 vs. 1.5 T scanners). Finally, to evaluate the ability of aMSM to identify trends in atrophy across a range of sample sizes, we performed four parallel analyses using diagnostic groups made up of 12, 24, 48, and 90 subjects per group.

## 2 MATERIALS AND METHODS

### 2.1 Data

Data used in the preparation of this article were obtained from the ADNI database (adni.loni.usc.edu). ADNI was launched in 2003 as a public–private partnership, led by Principal Investigator Michael W. Weiner, MD. The primary goal of ADNI has been to test whether serial MRI, positron emission tomography (PET), other biological markers, and clinical and neuropsychological assessment can be combined to measure the progression of MCI and early AD. For up‐to‐date information, see www.adni-info.org.

### 2.2 Subject selection

All subjects who participated in ADNI1 and underwent both baseline and 2‐year 3.0 T MRI scans were considered for inclusion in the primary analysis of this study. Age of subjects at the beginning of the study ranged from 55 to 90. All participants were required to have a study partner able to independently evaluate their functioning, be fluent in either English or Spanish, and be willing and able to participate in testing procedures and longitudinal follow‐up.

In ADNI1, cognitively normal subjects were defined as having Mini‐Mental State Exam (MMSE; Folstein, Folstein, & McHugh, 1975) scores ranging from 24 to 30 and a Clinical Dementia Rating (CDR; Morris, 1993) of 0. They were not depressed and did not suffer from MCI or dementia. MCI subjects had MMSE scores ranging from 24 to 30, a memory complaint, memory loss as measured on Wechsler Memory Scale Logical Memory II (Wechsler, 1987), a CDR of 0.5, and minimal impairment in other cognitive domains. Subjects in the mild AD group had MMSE scores ranging from 20 to 26, a CDR of 0.5 or 1.0, and met National Institute of Neurological and Communicative Disorders and Stroke‐Alzheimer's Disease and Related Disorders Association (NINCDS‐ADRDA; McKhann et al., 1984) criteria for probable AD.

To ensure that all subjects were scanned on the same scanner at baseline and 2‐year follow‐up, only subjects from standardized analysis sets were considered (Wyman et al., 2013). From this cohort, we identified subjects with baseline and 2‐year 3.0 T scans. Subjects were removed from analysis if their baseline or 2‐year scan failed at any point in MRI processing, including segmentation or registration. To test for significant differences in age, education, gender, race, ethnicity, study site, and MRI scanner manufacturer between diagnostic groups, one‐way ANOVA or the exact multinomial test of goodness‐of‐fit were used as appropriate.

While our primary analysis focuses on 3.0 T MRI scans, ADNI1 1.5 T scans were utilized to further validate our methodology. In ADNI1, only a quarter of subjects were scanned at 3.0 T, while all subjects were scanned on 1.5 T MRI scanners. This provided a large pool of additional subjects to supplement our primary, small cohort. ADNI1 subjects included in the standardized analysis sets (Wyman et al., 2013) who had baseline and 2‐year follow‐up 1.5 T scans were considered for this analysis. Subjects were excluded if MRI processing failed at any point, including segmentation or registration.

### 2.3 MRI acquisition and segmentation

MRI scans were collected according to the ADNI1 MRI protocol, which included back‐to‐back 3D magnetization prepared rapid gradient echo (MP‐RAGE) scans and B_1_ calibration scans when applicable (Jack et al., 2008). To increase standardization of images taken at different sites, postacquisition system‐specific corrections were performed to address image artifacts (Jack et al., 2008). Corrected artifacts include image geometry, intensity nonuniformity, and image intensity (Jack et al., 2008).

Cortical reconstruction and volumetric segmentation were performed with FreeSurfer, version 6.0.0, as described in prior publications (Dale, Fischl, & Sereno, 1999; Dale & Sereno, 1993; Fischl et al., 2002; Fischl et al., 2004; Fischl et al., 2004; Fischl & Dale, 2000; Fischl, Liu, & Dale, 2001; Fischl, Sereno, & Dale, 1999; Fischl, Sereno, Tootell, & Dale, 1999; Han et al., 2006; Ségonne et al., 2004). Briefly, the FreeSurfer pipeline accomplishes motion correction, skull stripping, Talairach transformation, segmentation of gray and white matter, intensity normalization, and tessellation of the gray‐white matter boundary. FreeSurfer also produces cortical thickness maps based on information derived from segmentation and deformation procedures (Fischl & Dale, 2000).

We utilized quality assurance images produced by the ciftify cifti_vis_recon_all utility (Dickie et al., 2019) to conduct an initial review of segmentation quality. In a minority of cases, segmentation quality could not be determined from these images alone. In these instances, midthickness surfaces were opened in Connectome Workbench (Marcus et al., 2011) and manually reviewed. Scans were excluded from analysis if automated segmentation did not correctly isolate the cerebral cortex.

### 2.4 Surface‐based registration

Ciftify (Dickie et al., 2019) was used to translate FreeSurfer output into a format consistent with the Human Connectome Project (HCP) pipeline (Glasser et al., 2013) and to accomplish registration to the MNI space. Surfaces from the native space, as defined in the HCP pipeline, were used as the starting point for longitudinal analysis with aMSM.

**FIGURE 1:**
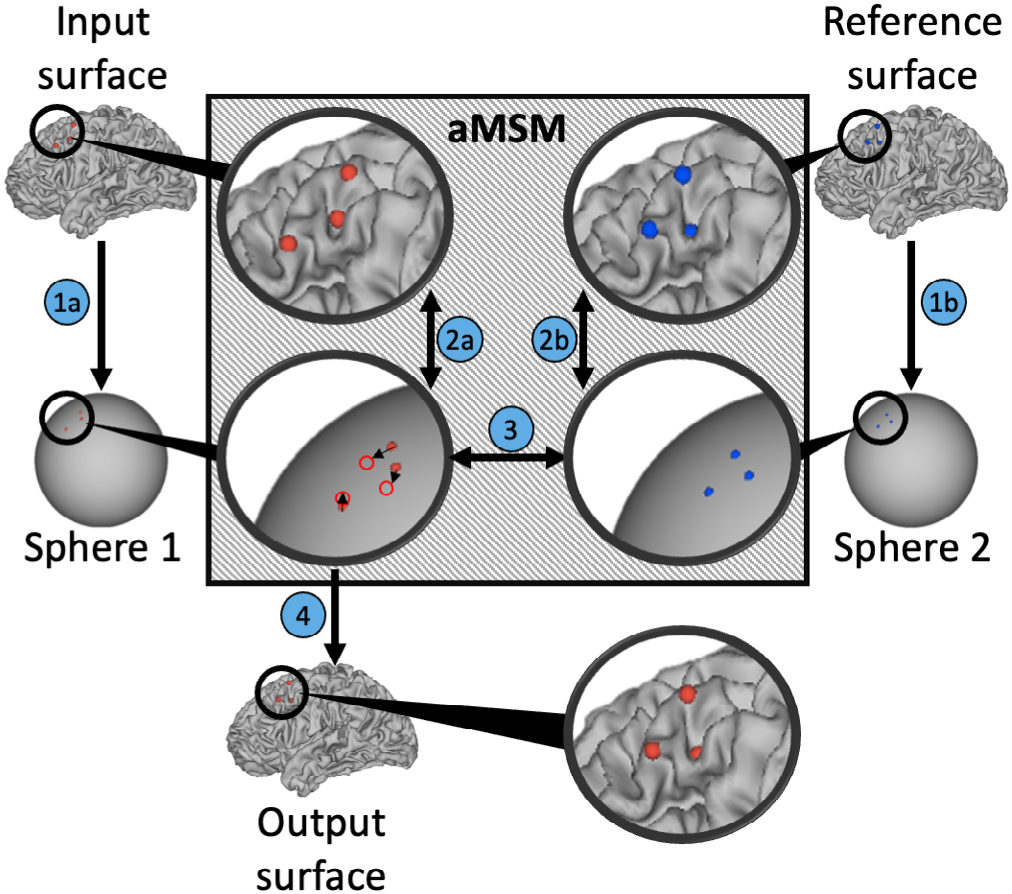
Longitudinal registration with aMSM. Physical deformation of the cortical surface between two timepoints is calculated for each triplet of vertices on the input surface (red dots) and reference surface (blue dots). Similar to other popular surface registration techniques (Yeo et al., 2010) vertices from both the input surface and reference surface are projected to a spherical representation to simplify mathematical calculations. However, unlike other registration techniques, aMSM (gray box) regularizes vertex displacement to minimize physical deformation (strain energy based on mechanical properties of brain tissue) between the anatomical surfaces (2a, 2b). Based on these constraints, triplets on sphere 1 are shifted to bring them into alignment with triplets on sphere 2 (3). Once realigned, sphere 1 is projected to a new anatomical output surface (4), resulting in more realistic physical deformations of the cortical surface

aMSM was used to align each subject's baseline and 2‐year cortical reconstructions (Robinson et al., 2014; Robinson et al., 2018). As shown in Figure 1, aMSM modifies an input surface, bringing it into alignment with a reference surface and producing an output surface that has point‐correspondence to the reference surface. Each surface can be represented as a series of vertices that connect to form triangular faces. aMSM evaluates these triangular faces to calculate cortical deformations that exist between the input and reference surfaces, while penalizing deformations incongruent with the mechanical properties of brain tissue (Garcia et al., 2018). Uniquely, aMSM penalizes these deformations on the anatomical surface (Figure 1), offering increased accuracy over alternatives relying on deformations of the spherical surface, which introduce additional artifact (Robinson et al., 2018). After registration, the deformations of each triangle provide detailed, local measures to map change in cortical surface area at the resolution of the control point grid (10,242 vertices in this study, corresponding to an average resolution of approximately 20 mm^2^).

Each subject's left and right hemispheres were processed separately, and each hemisphere was processed bidirectionally. Bidirectional maps of cortical change, one produced by registration of baseline surface to 2‐year surface and the other by registration of 2‐year surface to baseline surface, were averaged to minimize potential bias associated with unidirectional registration (Garcia et al., 2018). Each aMSM job ran on a single processor, and multiple jobs were run in parallel as computing resources allowed. The median runtime for aMSM was 26.08 hr, with a runtime of <36 hr in 92% of cases.

### 2.5 Global and local measures of atrophy

The primary measures considered in this study were change in cortical thickness, change in surface area, and change in volume. For each subject, change in cortical thickness was calculated as log_2_(CT_2_/CT_1_), where CT_1_ refers to cortical thickness at baseline and CT_2_ refers to cortical thickness at 2‐year follow‐up. Both CT_1_ and CT_2_ are FreeSurfer‐based thickness measurements. Individual thickness maps were smoothed using a 20 mm full‐width at half‐maximum (FWHM) Gaussian kernel, in accordance with past studies (Bernal‐Rusiel, Atienza, & Cantero, 2010; Kälin et al., 2017; Kang et al., 2019; Weston et al., 2016).

While local thickness can be defined for each vertex, local surface area is calculated from the area forming a triangle between each triplet of vertices. To obtain a vertex‐wise representation of local surface area, each vertex was assigned one third of the area for each triangle of which it is a part (Marcus et al., 2011). Change in cortical surface area was calculated as log_2_(SA_2_/SA_1_), where SA_1_ refers to surface area attributed to a given vertex at baseline and SA_2_ refers to surface area attributed to a given vertex at 2‐year follow‐up. Change in surface area was calculated by aMSM, with the final metric representing the averaged results from each unidirectional registration. Change in cortical volume was calculated as log_2_((SA_2_ × CT_2_)/(SA_1_ × CT_1_)).

To identify changes occurring at the global level, percent change in thickness, surface area, and volume were calculated at baseline and at 2‐year follow‐up for each subject. Vertices associated with the medial wall were excluded from all calculations. For a global measure of change in thickness, thickness was averaged across all cortical vertices. For a global measure of change in surface area, midthickness surface area was summed across all cortical vertices. Total volume was estimated by multiplying average thickness by total surface area. Percent change was defined as the difference between baseline and 2‐year follow‐up divided by the baseline result.

### 2.6 Statistics

To evaluate atrophy at the global level, one‐way ANOVA was used to compare measures of atrophy between demographically balanced diagnostic groups. Tukey's Honest Significant Difference test was used to conduct pairwise comparisons. Effect size was also calculated, as Cohen's *d*, for each pair of diagnostic groups that differed significantly in terms of change in thickness, surface area, or volume. *p* values and effect size are reported parenthetically in the text as *p* and *d*, respectively.

Statistical analysis of continuous surface maps for the CN, MCI‐S, MCI‐C, and AD diagnostic groups was conducted using Permutation Analysis of Linear Models (PALM) with threshold‐free cluster enhancement and family‐wise error correction (Winkler, Ridgway, Webster, Smith, & Nichols, 2014). To find regions of significant atrophy in each diagnostic group, one‐sample *t*‐tests were performed separately for each group. ANOVA was performed to determine regions of intergroup differences in atrophy. All possible pairwise comparisons were analyzed, and all analyses were performed on surfaces with 10,242 vertices per hemisphere.

### 2.7 FreeSurfer longitudinal pipeline

The FreeSurfer longitudinal pipeline is a commonly used surface‐based method of measuring cortical atrophy. Like aMSM, the FreeSurfer longitudinal pipeline accomplishes longitudinal registration, allowing for the measurement of structural cortical change within a single subject over time. To directly compare these two registration methods, we reanalyzed the CN, MCI‐S, MCI‐C, and AD diagnostic groups using the FreeSurfer longitudinal pipeline instead of aMSM.

The FreeSurfer surfaces described in the “MRI acquisition and segmentation” section were used as a starting point for processing with the FreeSurfer longitudinal pipeline. This pipeline produced aligned baseline and 2‐year follow‐up surfaces for each subject. These surfaces were processed with ciftify and changes in thickness, surface area, and volume were calculated for each subject as described in the “Global and local measures of atrophy” section. Once atrophy maps had been generated for each subject, statistical analyses were performed using PALM as described in the “Statistics” section.

### 2.8 Region of interest analysis

ROI analysis is a commonly used alternative to the continuous surface maps that are the focus of this study. To compare this methodology to results produced using aMSM, we reanalyzed atrophy in the CN, MCI‐S, MCI‐C, and AD diagnostic groups in terms of ROIs. The same FreeSurfer segmented and ciftify‐processed surfaces that were used in our primary analysis were used for ROI analysis.

ROIs were based on the Desikan‐Killiany (DK) protocol (Desikan et al., 2006). Within each ROI, average thickness and total surface area were calculated for each subject at both baseline and 2‐year follow‐up. Change in cortical thickness, surface area, and volume were calculated for each subject, at all ROIs, using the equations described in the “Global and local measures of atrophy” section.

One‐way multivariate ANOVA (MANOVA) was used to identify differences in atrophy between diagnostic groups. Separate MANOVA were run for change in cortical thickness, change in cortical surface area, and change in cortical volume. Left and right hemispheres were processed separately. Differences between specific groups were assessed using pairwise comparisons with Bonferroni correction.

### 2.9 Comparisons using 1.5 T Scans

To demonstrate the influence of MRI field strength on the measurement of atrophy, we identified subjects from our primary 3.0 T dataset who had baseline and 2‐year follow‐up scans performed on both 1.5 and 3.0 T scanners. Matching 1.5 and 3.0 T datasets were analyzed in parallel to identify the impact of field strength on changes in thickness, surface area, and volume. One‐sample *t*‐tests were performed using PALM, as described in the “Statistics” section, to define patterns of atrophy within each diagnostic group at each field strength.

We also used the larger cohort of ADNI1 subjects, scanned at 1.5 T, to examine the relative performance of surface area and thickness measures at a variety of sample sizes. Demographically balanced 90‐subject samples were selected for each diagnostic group. To test for significant differences between diagnostic groups in age, education, gender, race, ethnicity, study site, and MRI scanner manufacturer, one‐way ANOVA, the chi square test, or the exact multinomial test of goodness‐of‐fit were used as appropriate. To assess performance of 1.5 T scans at lower sample sizes, smaller samples of 12, 24, and 48 subjects per diagnostic group were randomly selected (Urbaniak & Plous, 2013) from within the 90‐subject samples. ANOVA, performed using PALM as described in the “Statistics” section, was used to identify differences in atrophy between diagnostic groups at each sample size.

## 3 RESULTS

### 3.1 Subjects

In total, 89 subjects met eligibility criteria for inclusion in our primary, 3.0 T analysis. Of those subjects, 18 were removed following postsegmentation quality review and another five failed to successfully complete longitudinal registration with aMSM. Of the 66 subjects remaining after MRI processing, an additional 16 were removed from analysis to demographically balance the four diagnostic groups. This left us with a relatively small cohort of 50 subjects, comparable to the small sample sizes in pilot studies or observational studies focused on specific AD populations or subtypes (Machado et al., 2020; Persson et al., 2017). Demographic characteristics of the CN, MCI‐S, MCI‐C, and AD groups are presented in Table 1. The manufacturers of the MRI machines used to scan this cohort are presented in Table 2, and the study sites visited by subjects in the four diagnostic groups are presented in Table S1.

**TABLE 1:** Subject demographics by diagnostic group in ADNI1 3.0 T cohort

Characteristic	CN	MCI‐S	MCI‐C	AD
Total subjects	12	12	13	13
Gender				
Males	6	6	8	4
Females	6	6	5	9
Ethnicity				
Hispanic or Latino	1	1	1	0
Not Hispanic or Latino	11	11	12	13
Race				
Black or African American	1	0	0	2
White	11	12	13	11
Mean age (*SD*) at baseline (years)	72.39 (1.93)	72.14 (6.65)	72.89 (9.62)	71.46 (8.32)
Mean (*SD*) years education	16.08 (1.62)	15.33 (2.35)	14.31 (3.95)	14.08 (3.82)

**TABLE 2:** MRI scanner manufacturers for ADNI1 3.0 T cohort

Manufacturer	CN	MCI‐S	MCI‐C	AD
GE medical systems	2	1	0	2
Philips medical systems	5	5	3	4
Siemens	5	6	10	7
Total	12	12	13	13

A total of 499 ADNI1 subjects met eligibility criteria for inclusion in our secondary, 1.5 T analysis. Of these, 16 subjects failed segmentation and four failed registration. Of the remaining 479 subjects, 160 were in the CN group, 127 in the MCI‐S group, 92 in the MCI‐C group, and 100 in the AD group. Forty‐four subjects, 11 in the CN group, nine in the MCI‐S group, 12 in the MCI‐C group, and 12 in the AD group, also had successfully processed 3.0 T scans. These 44 subjects were used, along with their 3.0 T counterparts, in our analysis of the influence of field strength on the measurement of atrophy.

For our analysis of the influence of sample size on the measurement of atrophy, the 479 successfully processed subjects with 1.5 T scans served as the pool for selecting demographically balanced 90‐subject samples within each diagnostic group. Demographic characteristics of these samples are presented in Table 3. The manufacturers of the MRI machines used to scan these subjects are presented in Table 4, and study sites visited are presented in Table S2. The 12, 24, and 48 subject samples used in our sample size analysis were randomly selected from within the samples presented in Table 3. Demographic and MRI scanner manufacturer information for the 12, 24, and 48 subject samples is presented in Tables S3–S8.

**TABLE 3:** Subject demographics by diagnostic group in 90 subject ADNI1 1.5 T samples

Characteristic	CN	MCI‐S	MCI‐C	AD
Total subjects	90	90	90	90
Gender				
Males	53	51	61	48
Females	37	39	29	42
Ethnicity				
Hispanic or Latino	2	2	1	0
Not Hispanic or Latino	88	88	89	89
Unknown	0	0	0	1
Race				
Asian	1	2	3	1
Black or African American	4	4	2	3
White	85	84	85	86
Mean age (*SD*) at baseline (years)	76.32 (5.36)	74.95 (6.00)	74.92 (7.42)	75.60 (7.33)
Mean (*SD*) years education*	15.70 (2.69)	15.13 (2.62)	15.61 (3.00)	14.86 (3.02)

**TABLE 4:** MRI scanner manufacturers for 90 subject ADNI1 1.5 T samples

Manufacturer	CN	MCI‐S	MCI‐C	AD
GE medical systems	48	54	48	45
Philips medical systems	5	3	8	12
Siemens	37	33	34	33
Total	90	90	90	90

### 3.2 Global atrophy

**FIGURE 2:**
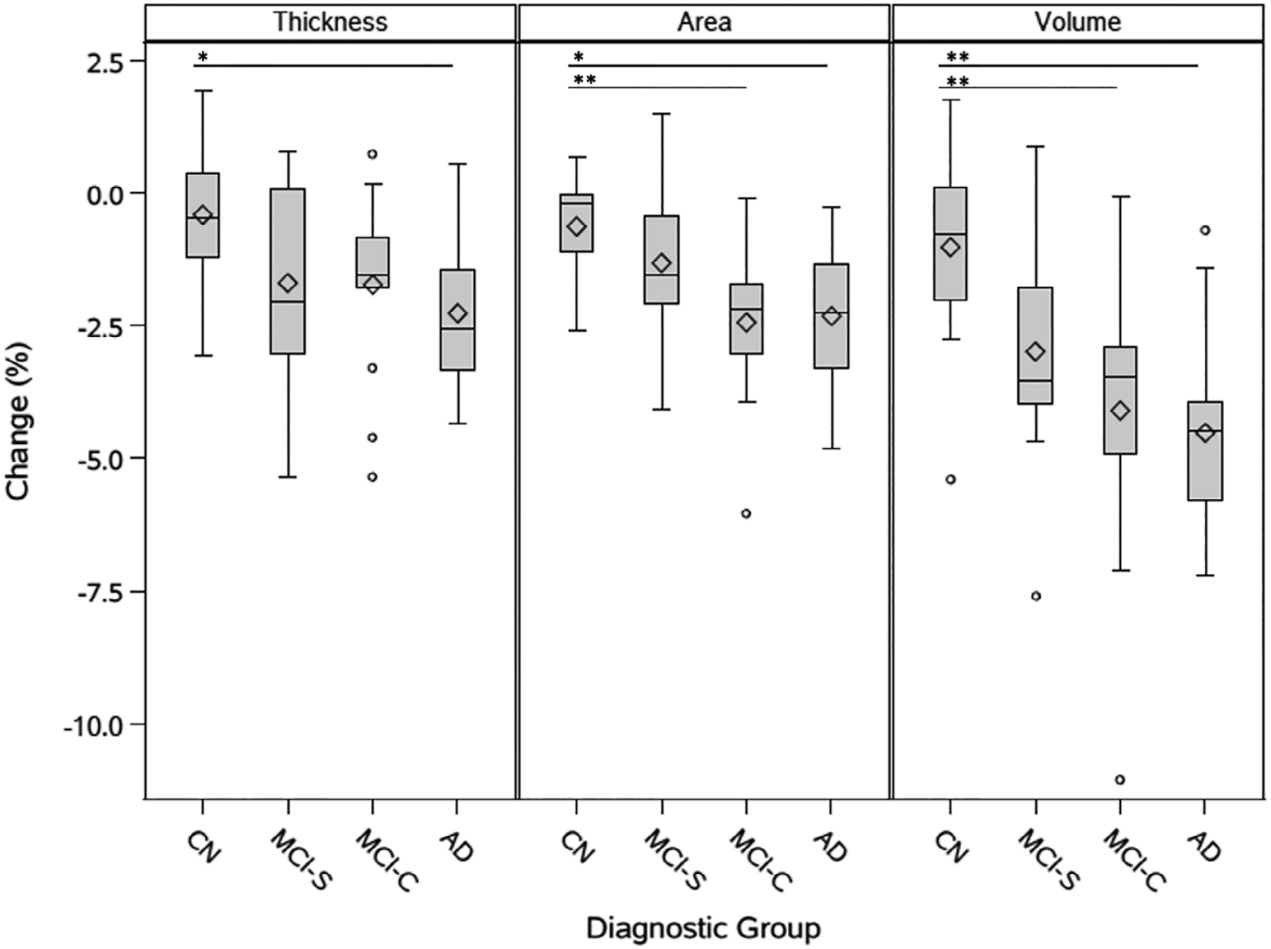
Global loss in thickness, surface area, and volume by diagnostic group. Differences between diagnostic groups (CN, MCI‐S, MCI‐C, AD) were identified by ANOVA. Significant differences with .01 ≤ *p* < .05 are identified with the symbol *. Very significant differences with *p* < .01 are identified with the symbol **. Diamond symbols indicate group means. Lower and upper box edges represent the first quartile (Q1) and third quartile (Q3), respectively, with the area within the box representing the interquartile range (IQR). Horizontal lines inside of boxes represent group medians. Whiskers extend to the minimum and maximum nonoutlier values, where outliers are defined as falling below a minimum value of (Q1 – 1.5*IQR) or above a maximum value of (Q3 + 1.5*IQR). Outliers are indicated with circles

Comparisons of global loss in thickness, surface area, and volume were conducted between the four diagnostic groups: CN, MCI‐S, MCI‐C, and AD. As shown in Figure 2, a significant difference in thickness was identified between the CN and AD groups (*p* = .034, *d* = 1.334), but not between the CN and MCI‐C groups (*p* = .199, *d* = 0.843) or the CN and MCI‐S groups (*p* = .232, *d* = 0.780). Significant differences in surface area were found between the CN and AD groups (*p* = .017, *d* = 1.366), as well as between the CN and MCI‐C groups (*p* = .009, *d* = 1.413), but not between the CN and MCI‐S groups (*p* = .605, *d* = 0.540). Similarly, significant differences in volume were found between the CN and AD groups (*p* = .002, *d* = 1.806), as well as between the CN and MCI‐C groups (*p* = .008, *d* = 1.253), but not between the CN and MCI‐S groups (*p* = .168, *d* = 0.946). Of the three measures considered, change in volume identified the most pronounced differences between groups.

### 3.3 Individual atrophy maps

**FIGURE 3:**
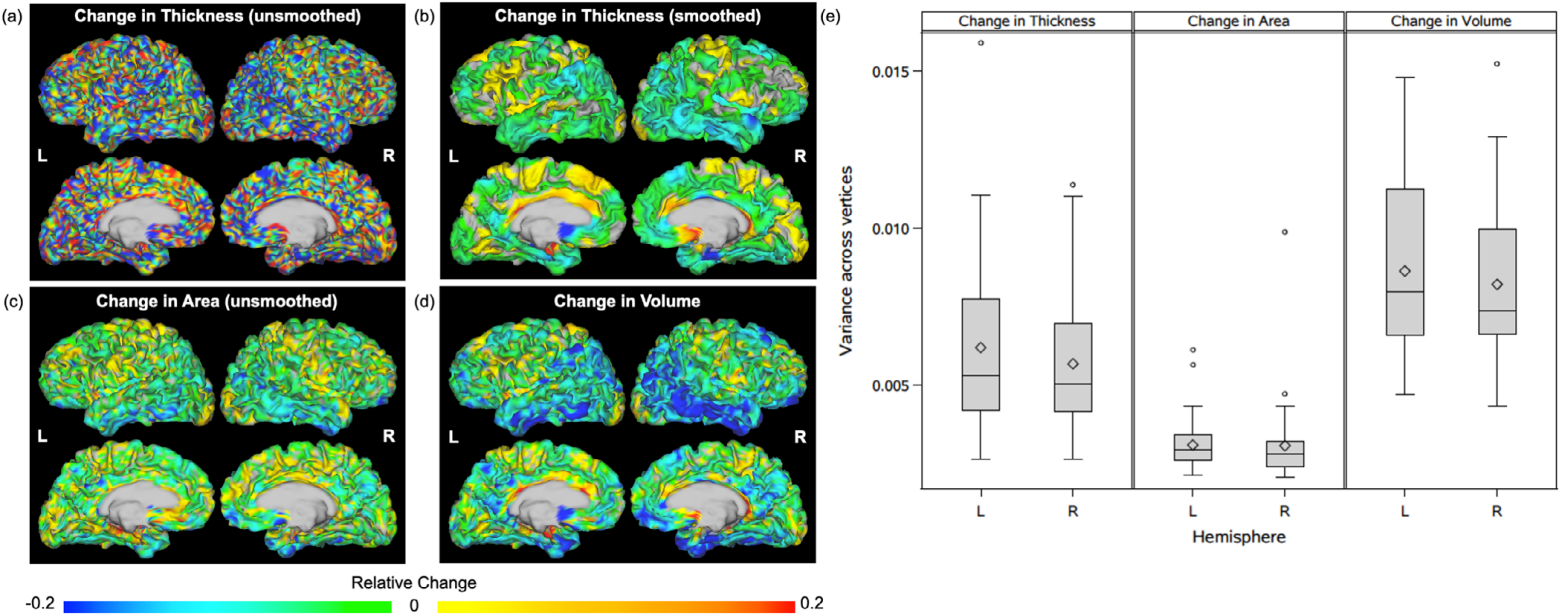
Change in unsmoothed thickness, smoothed thickness, surface area, and volume within individual subjects. Relative change in (a) unsmoothed thickness, (b) smoothed thickness, (c) surface area, and (d) volume for a single subject in the AD group. Change in volume is calculated using smoothed thickness and unsmoothed surface area maps. Panel (e) demonstrates intrasubject variance in smoothed thickness (left), surface area (middle), and volume (right) change across all 50 study subjects. Results are demonstrated for the left (L) and right (R) hemispheres. Diamond symbols indicate group means. Lower and upper box edges represent the first quartile (Q1) and third quartile (Q3), respectively, with the area within the box representing the interquartile range (IQR). Horizontal lines inside of boxes represent group medians. Whiskers extend to the minimum and maximum nonoutlier values, where outliers are defined as falling below a minimum value of (Q1 − 1.5*IQR) or above a maximum value of (Q3 + 1.5*IQR). Outliers are indicated with circles

In order to identify patterns of atrophy within each diagnostic group, we first calculated individual atrophy maps for each subject. Figure 3 demonstrates maps of change in unsmoothed thickness, smoothed thickness, surface area, and volume for one subject in the AD group. Maps of unsmoothed thickness (Figure 3a) contained considerable noise, while maps of smoothed thickness (Figure 3b) demonstrated biologically feasible patterns of atrophy, illustrating the rationale for using smoothed thickness for group‐based analysis and calculation of cortical volume. The map for change in surface area (Figure 3c), produced using aMSM, provides a novel measure of longitudinal structural change. Change in volume (Figure 3d), which incorporates both change in smoothed thickness and change in surface area, demonstrates the greatest degree of atrophy. Figure 3e illustrates intrasubject variance associated with change in smoothed thickness, change in surface area, and change in volume. Within each subject, standard deviation was calculated for each measure using that measure's value at every vertex, excluding the medial wall. Standard deviation was then used to calculate variance. Figure 3e summarizes the variability of each metric (variance of all vertex values for a given subject) for all 50 subjects. The variability across vertices was lowest when considering change in surface area.

### 3.4 Patterns in atrophy by diagnostic group

**FIGURE 4:**
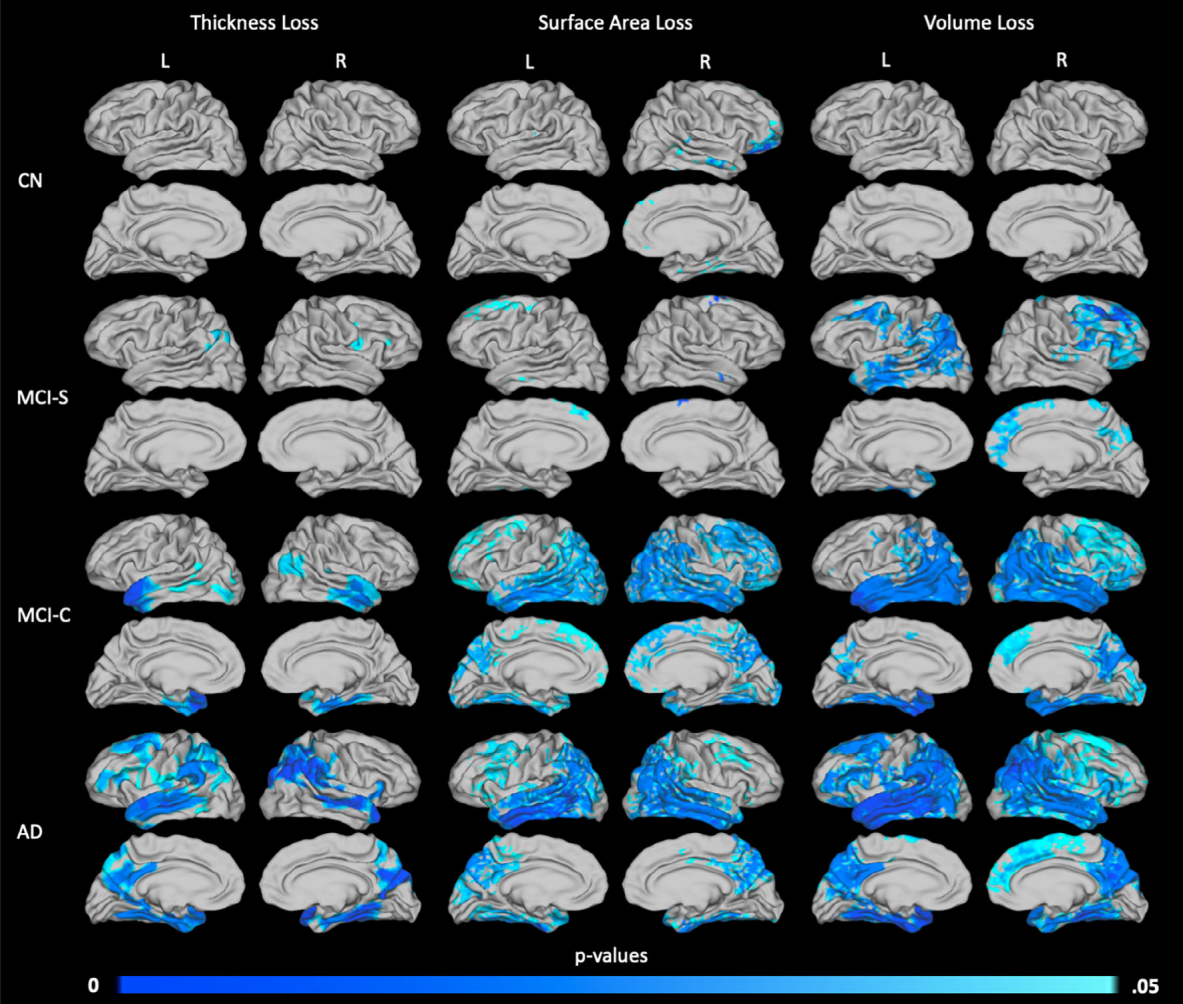
Areas of significant thickness, surface area, and volume loss over a 2‐year period by diagnostic group. Patterns of atrophy are shown for the CN (*n* = 12, top row), MCI‐S (*n* = 12, second row), MCI‐C (*n* = 13, third row), and AD (*n* = 13, bottom row) groups, in terms of loss in thickness (left column), surface area (middle column), and volume (right column). *p* values are threshold‐free cluster‐enhanced with family‐wise error correction

Within each diagnostic group, patterns associated with change in cortical thickness, surface area, and volume were identified using one‐sample *t*‐tests (Figure 4). Across all metrics, relatively little atrophy was identified in the CN and MCI‐S groups, but considerable atrophy was present in the more symptomatic MCI‐C and AD groups. These latter groups showed particularly strong patterns of atrophy in the temporal and parietal lobes, though atrophy was detected throughout much of the cortex. For all groups except CN, the greatest degree of atrophy was detected by volume loss, which combines thickness and surface area loss.

Surface area and thickness loss identified similar regions of atrophy. However, the specific patterns and degrees of atrophy detected by each measure differed. Significant decreases in surface area tended to be detected over broader regions of cortex, while decreases in thickness were more localized. There were also some differences in the specific regions of atrophy identified by these two measures. For example, in the MCI‐C group (Figure 4, row 3), significant loss of surface area was detected in the frontal lobe, where no significant loss was detected in terms of thickness. Conversely, the anterior portion of the left superior temporal gyrus displays significant thickness loss despite little evidence of loss in surface area. Comparisons of volume loss showed the greatest significance, even suggesting cortical loss in specific areas of the MCI‐S group.

### 3.5 Areas of increased atrophy in the AD and MCI‐C diagnostic groups

**FIGURE 5:**
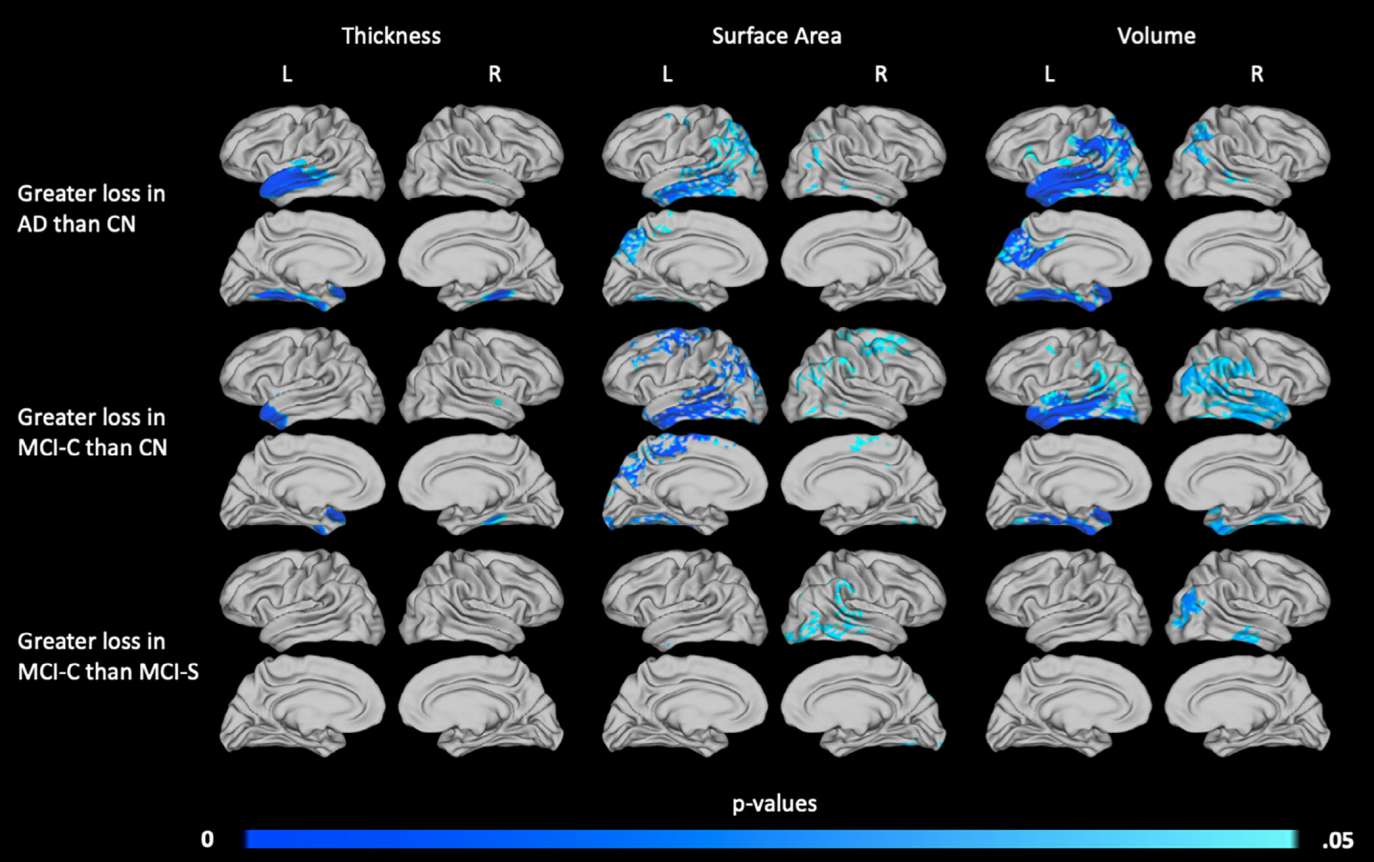
Areas of increased 2‐year atrophy in the AD and MCI‐C diagnostic groups relative to the CN and MCI‐S groups. Diagnostic groups were compared to identify differences in patterns of atrophy, and significant differences were found between AD and CN groups (top row), MCI‐C and CN groups (middle row), and MCI‐C and MCI‐S groups (bottom row). Areas in which thickness (left column), surface area (middle column), or volume (right column) loss differ are shown. *p* values are threshold‐free cluster‐enhanced with family‐wise error correction

To compare differences in atrophy among the four diagnostic groups, ANOVA was used to compare groups within each atrophy metric (loss in thickness, surface area, or volume). Divergent patterns of atrophy between the AD and CN groups, the MCI‐C and CN groups, and the MCI‐C and MCI‐S groups are illustrated in Figure 5. The AD and MCI‐S groups also showed different patterns of atrophy, but only in the right hemisphere. These differences are similar to those seen in the right hemisphere when comparing the AD and CN groups (Figure 5, top row). No other pairwise comparisons demonstrated significant differences in atrophy. In all comparisons that did identify significant differences in atrophy, additional regions of atrophy were detected in the AD and MCI‐C groups that were not present in the CN and MCI‐S groups.

As shown in Figure 5, atrophy maps produced using thickness loss and surface area loss identified distinct areas of difference between groups, with little overlap. For example, when comparing the AD and CN groups (Figure 5, top row), the AD group shows significant thickness loss, but not surface area loss, in the left superior temporal gyrus. The same group shows surface area loss in the left inferior temporal gyrus, but no thickness loss at this location. Differences between thickness‐ and surface area‐derived maps are also distinct when comparing the MCI‐C and CN groups (Figure 5, middle row). The MCI‐C group displayed increased loss in thickness almost exclusively in the left anterior temporal pole, while increased surface area loss was evident in the bilateral temporal, parietal, and primary/premotor cortices.

Overall, volume loss was again the most sensitive measure analyzed, identifying larger and more significant areas of difference than thickness or surface area loss alone. In the comparison of the AD and CN groups, results were more significant when volume was considered. The map of change in volume resulting from this comparison combines most areas of atrophy associated with either thickness or surface area loss. The same trend can be seen in the comparison of the MCI‐C and CN groups. Finally, when comparing the MCI‐C and MCI‐S groups, volume loss identified areas of atrophy with greater significance than either thickness loss or surface area loss alone.

### 3.6 Cortical atrophy measured with aMSM, the FreeSurfer longitudinal pipeline, and ROIs

To highlight the strengths and limitations of aMSM, we compared results obtained using aMSM to two additional methodologies: vertex‐wise analysis based on longitudinal registration using the FreeSurfer longitudinal pipeline and ROI analysis based on the DK atlas.

**FIGURE 6:**
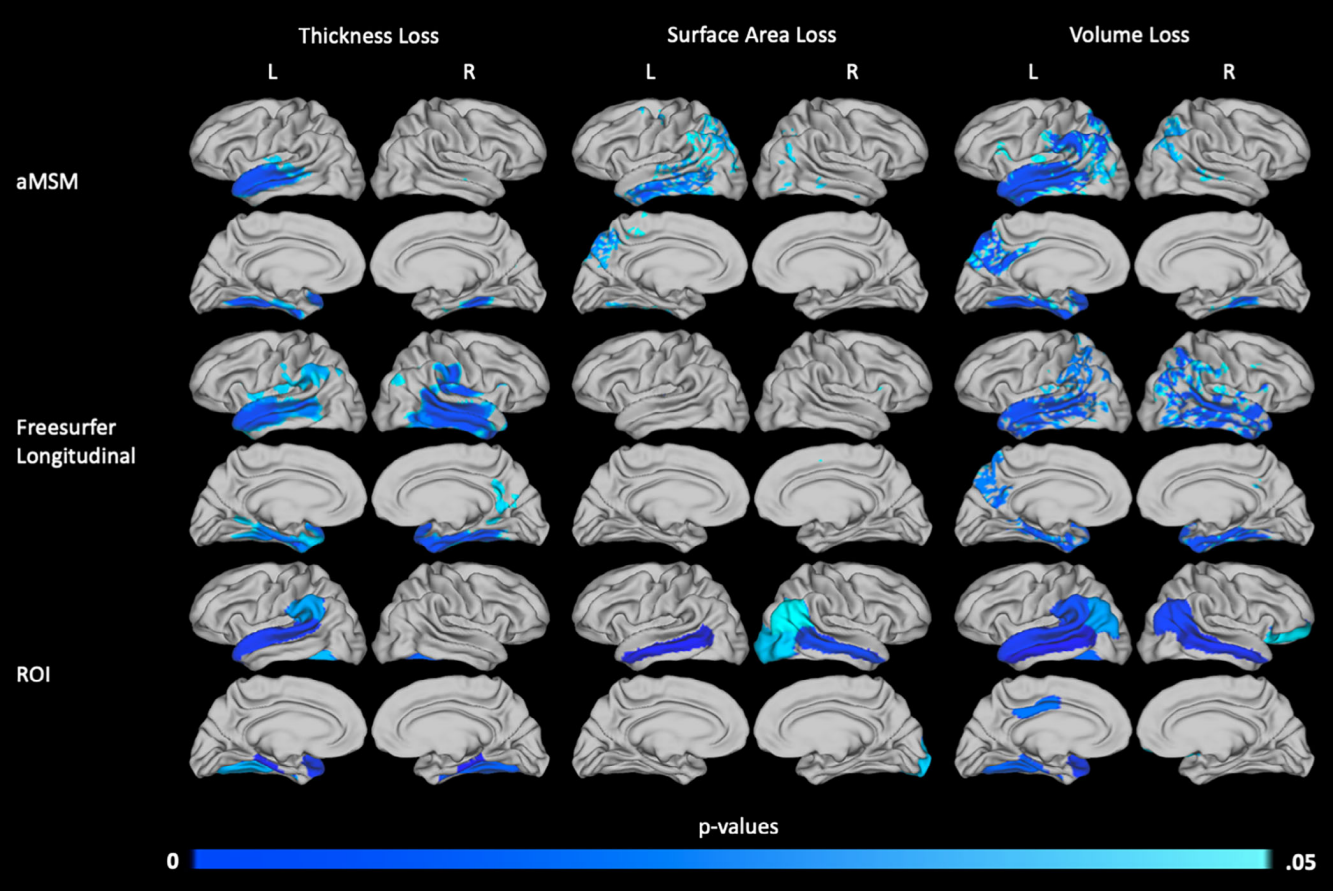
Areas of increased 2‐year atrophy in the AD group relative to the CN group, calculated using aMSM, the FreeSurfer longitudinal pipeline, and predefined ROIs. Areas in which thickness (left column), surface area (middle column), or volume (right column) loss differ between the AD and CN groups are shown. *p* values for the continuous surface maps produced using aMSM (top row) and the FreeSurfer longitudinal pipeline (middle row) are threshold‐free cluster enhanced with family‐wise error correction. *p* values associated with specific ROIs (bottom row) were adjusted with Bonferroni correction

**FIGURE 7:**
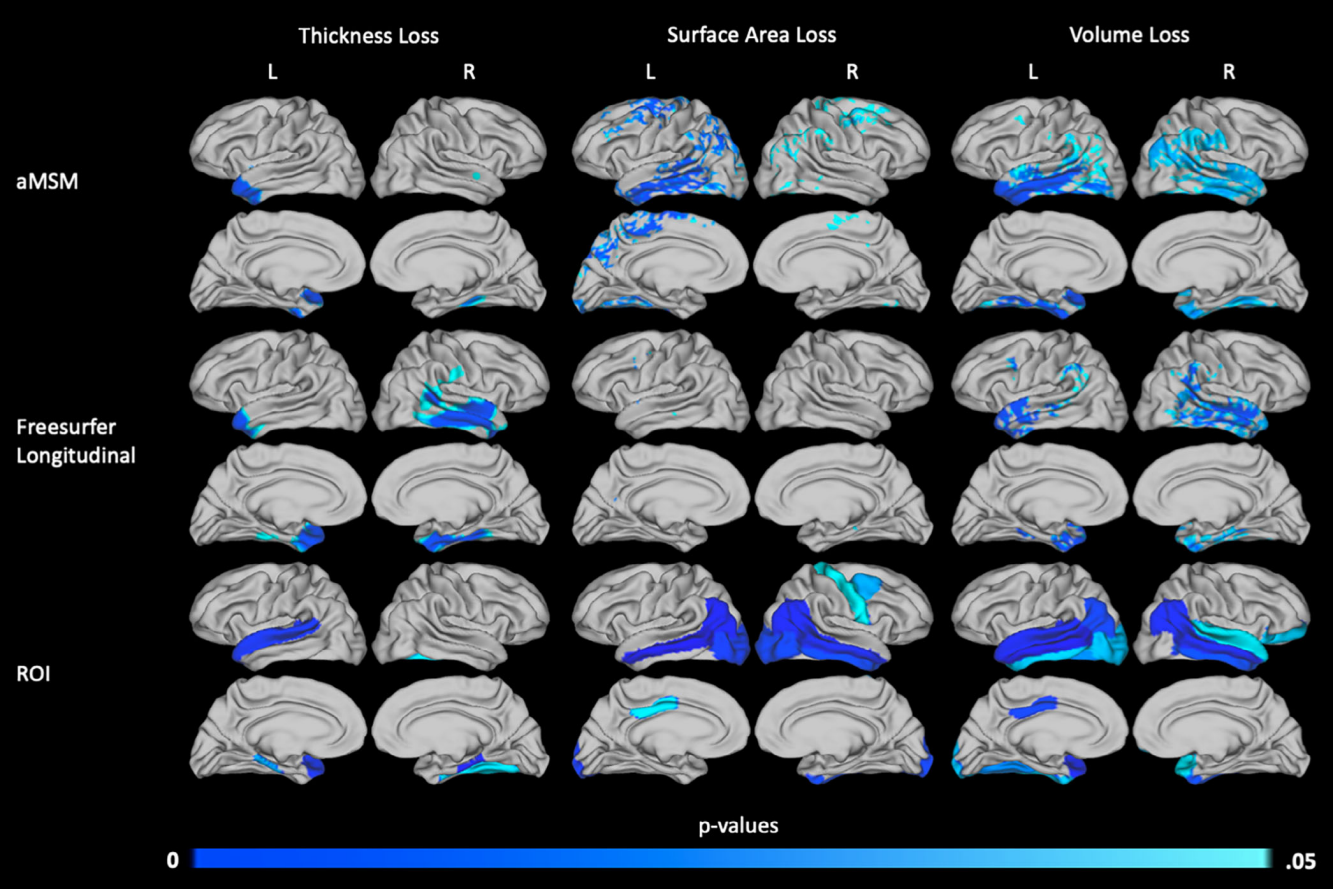
Areas of increased 2‐year atrophy in the MCI‐C group relative to the CN group, calculated using aMSM, the FreeSurfer longitudinal pipeline, and predefined ROIs. Areas in which thickness (left column), surface area (middle column), or volume (right column) loss differ between the MCI‐C and CN groups are shown. *p* values for the continuous surface maps produced using aMSM (top row) and the FreeSurfer longitudinal pipeline (middle row) are threshold‐free cluster enhanced with family‐wise error correction. *p* values associated with specific ROIs (bottom row) were adjusted with Bonferroni correction

Results of two illustrative pairwise comparisons, the AD and CN groups and the MCI‐C and CN groups, are shown in Figures 6 and 7, respectively. Overall, ROI results detected the most atrophy across metrics. However, conformity to predefined ROIs did, in some instances, distort the localization of cortical atrophy. For example, both aMSM and the FreeSurfer longitudinal pipeline identified the left temporal pole as a region of increased thickness loss in the MCI‐C group (Figure 7). However, due to the ROI definitions used, ROI analysis suggests that the entire superior temporal gyrus is an area of increased atrophy.

aMSM and the FreeSurfer longitudinal pipeline identified similar patterns of overall atrophy, as measured by change in volume. However, the influence of thickness and surface area differed between the two methodologies. aMSM was more sensitive than the FreeSurfer longitudinal pipeline to changes in cortical surface area, while the FreeSurfer longitudinal pipeline identified more areas of significant thickness loss. When each diagnostic group was evaluated individually for evidence of atrophy, as presented in Figures S1–S4, these trends persisted. Notably, surface area and thickness changes detected by aMSM appeared generally more similar to results detected by ROI analysis than did changes detected by the FreeSurfer longitudinal pipeline.

### 3.7 Influence of field strength on measurements of atrophy

**FIGURE 8:**
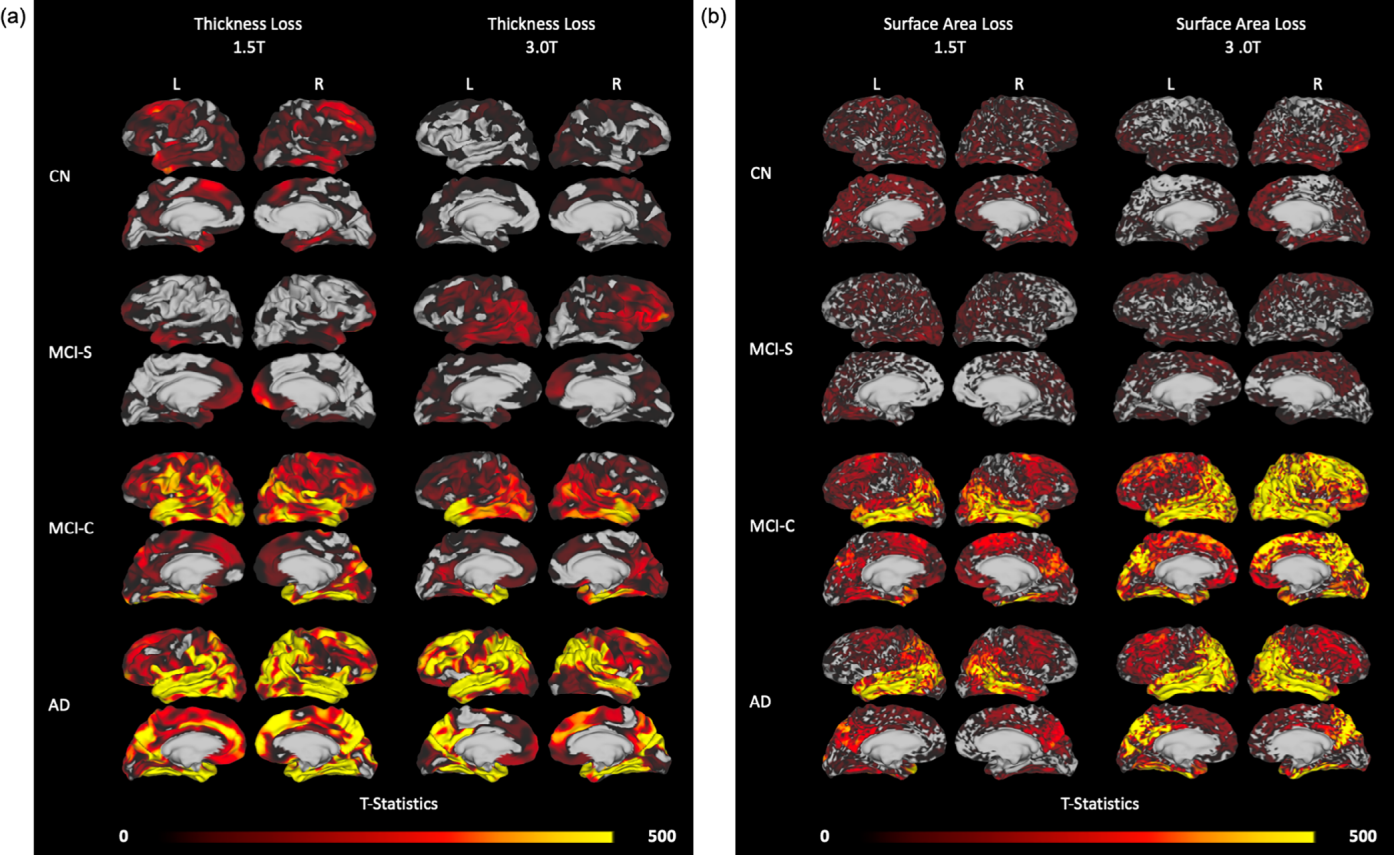
Areas of atrophy over a 2‐year period assessed in the same subjects with 1.5 and 3.0 T MRI. (a) Patterns of thickness loss in CN (*n* = 11), MCI‐S (*n* = 9), MCI‐C (*n* = 12), and AD (*n* = 12) groups. (b) Patterns of surface area loss in the same groups of subjects. Each panel displays atrophy patterns that were derived from 1.5 T MRI scans (left column) and 3.0 T scans (right column) of the same subjects over the same 2‐year period. *T*‐statistics are threshold‐free cluster enhanced

For a subset of the ADNI1 cohort, subjects were scanned using both 1.5 and 3.0 T acquisition parameters at baseline and 2‐year time points. To explore aMSM's ability to consistently and reliably measure atrophy under different image acquisition parameters, we analyzed and compared both sets of imaging data. The resulting maps of change in thickness (Figure 8a) and change in surface area (Figure 8b) illustrate patterns of atrophy detected using 1.5 and 3.0 T field strengths.

Overall, change in surface area was less impacted than change in thickness by differences in field strength. Loss in surface area was detected in the same regions of the cortex regardless of field strength, though there was generally more evidence of atrophy in the 3.0 T analysis. By contrast, thickness‐based atrophy performed less consistently. While some common trends were detected in both the 1.5 and 3.0 T analysis, a considerable amount of variation also existed. Maps of change in volume (Figure S5) identified more atrophy than change in surface area or change in volume alone but, due to variation in the thickness measure, did not measure atrophy as consistently as did change in surface area.

### 3.8 Influence of sample size on measurements of atrophy

**FIGURE 9:**
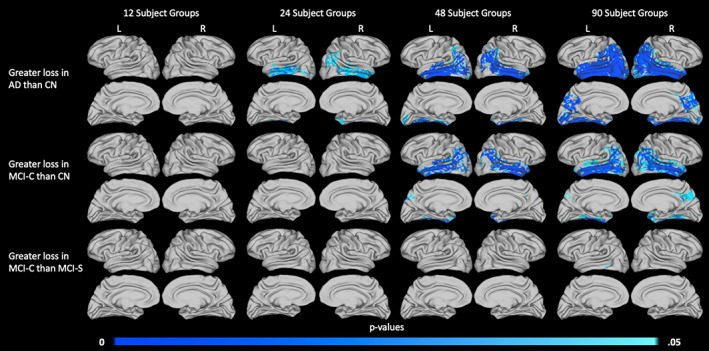
Influence of sample size on the detection of surface area loss. Significant differences in 2‐year atrophy between the AD and CN groups (top row), MCI‐C and CN groups (middle row), and MCI‐C and MCI‐S groups (bottom row) are displayed. Results are shown for 12 subjects per diagnostic group (column 1), 24 subjects per diagnostic group (column 2), 48 subjects per diagnostic group (column 3), and 90 subjects per diagnostic group (column 4). All subjects were scanned at 1.5 T. *p* values are threshold‐free clusterenhanced with family‐wise error correction

**FIGURE 10:**
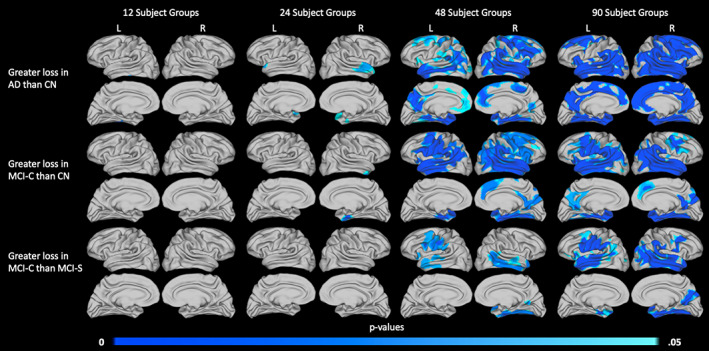
Influence of sample size on the detection of thickness loss. Significant differences in 2‐year atrophy between the AD and CN groups (top row), MCI‐C and CN groups (middle row), and MCI‐C and MCI‐S groups (bottom row) are displayed. Results are shown for 12 subjects per diagnostic group (column 1), 24 subjects per diagnostic group (column 2), 48 subjects per diagnostic group (column 3), and 90 subjects per diagnostic group (column 4). All subjects were scanned at 1.5 T. *p* values are threshold‐free clusterenhanced with family‐wise error correction

Finally, to demonstrate the reliability of aMSM results obtained using different sample sizes, we considered subsets of the large 1.5 T dataset. The largest sample considered is described in Table 3, with 90 subjects in each diagnostic group. Smaller samples of 12, 24, and 48 subjects per diagnostic group were randomly selected from within the large 1.5 T cohort. ANOVA was performed to identify trends in surface area loss (Figure 9), thickness loss (Figure 10), and volume loss (Figure S6) at each of the four sample sizes. The group comparisons depicted in Figures 9, 10, and S6 mirror those seen in Figure 5, which focused on the small 3.0 T cohort.

Our results suggest that measures of surface area may perform slightly better at small sample sizes than measures of thickness. Particularly, in the comparison of the AD and CN groups (Figure 9, top row), analysis of surface area loss in the 24‐ and 48‐subject groups identified very similar trends. These results also aligned well with trends seen in the 90‐subject groups. When examining areas of increased thickness loss (Figure 10, top row), only minimal evidence of increased atrophy was detectable in the 24‐subject groups.

When comparing the MCI‐C and CN groups (Figures 9 and 10, middle row) or the MCI‐C and MCI‐S groups (Figures 9 and 10, bottom row), surface area and thickness measures performed similarly. In both of these comparisons, minimal significant differences were identified in groups of fewer than 48 subjects, and 48‐ and 90‐subject group comparisons identified similar trends, including some substantial areas of atrophy. It is likely that few significant differences were identified between the 24‐subject groups because the overall effect sizes were smaller than those seen in the AD and CN comparison (Figures S7 and S8).

Notably, our primary analysis of subjects scanned at 3.0 T was able to detect significant differences in atrophy between groups when considering 12–13 subjects per group (Figure 5). However, we were unable to detect such differences between the 12‐subject 1.5 T groups (Figures 9 and 10). Trends that were detected in the small 3.0 T groups did tend to align with areas of increased atrophy in the larger 1.5 T groups, with even broader regions reaching statistical significance in the largest 1.5 T groups.

## 4 DISCUSSION

This study compared three measures of cortical atrophy: thickness loss, surface area loss, and volume loss. By utilizing aMSM for longitudinal registration, we produced maps of change in surface area, optimized with anatomically constrained deformations, which offer advantages over maps produced by commonly used spherically based methodologies (Robinson et al., 2018) and provide detail that cannot be achieved by global or ROI‐based analyses.

To illustrate the benefit of this method relative to other existing approaches, we compared maps produced with aMSM to two common alternatives, the FreeSurfer longitudinal pipeline and ROI analysis. Furthermore, to characterize the reliability of atrophy metrics derived from thickness, surface area, or volume, we examined the impact of MRI field strength and sample size on aMSM‐derived atrophy maps.

### 4.1 Surface area loss as a complementary measure of cortical atrophy

Our results suggest that mapping change in surface area is a viable method of detecting atrophy, with group analysis producing results of comparable significance to those detected using change in thickness (Figures 4 and 5). These similar levels of significance were detected despite the fact that surface area maps did not undergo smoothing procedures that were applied to thickness maps.

Overall, the patterns of atrophy detected with aMSM are consistent with prior studies. Areas of increased atrophy identified in AD include medial temporal lobe structures (Belathur Suresh et al., 2018; Chandra et al., 2019; Cuingnet et al., 2011; Femminella et al., 2018; Risacher et al., 2010), the lateral temporal lobe (Belathur Suresh et al., 2018; Chandra et al., 2019; Cuingnet et al., 2011; Femminella et al., 2018), the parietal cortex (Belathur Suresh et al., 2018; Chandra et al., 2019), the posterior cingulate (Cuingnet et al., 2011), and the precuneus (Belathur Suresh et al., 2018). Our comparison of MCI‐C and CN subjects also produced results consistent with past studies, which have found increased atrophy in medial temporal lobe structures (Chandra et al., 2019; Risacher et al., 2010) and the parietal and temporal cortices (Risacher et al., 2010) of MCI‐C participants.

Unsurprisingly, in this study we found the most sensitive measure of atrophy to be volume loss, which incorporates both surface area loss and thickness loss. Volume loss identified atrophy with the greatest degree of significance, whether considering global atrophy, individual subject vertex‐wise atrophy, intragroup patterns of atrophy, or intergroup differences in atrophy. However, as a multidimensional measure incorporating both change in thickness and change in surface area, change in volume masks the distinct influences of these two underlying measures.

It has been found that cortical surface area and thickness are both heritable traits with separate genetic influences (Grasby et al., 2020; Panizzon et al., 2009), and the two measures are not equally impacted as volume is lost in AD. In medial temporal lobe structures specifically, surface area loss is thought to be characteristic of normal aging, while AD is associated with additional significant thickness loss (Dickerson et al., 2009). Further, thinning of the entorhinal and perirhinal cortices was found to correlate with memory performance, while decreases in surface area did not (Dickerson et al., 2009). The results of our study have also demonstrated that patterns of thickness and surface area loss in AD differ, in the medial temporal lobe and in other regions of the cortex (Figure 5), though the clinical consequences of these differences require further study.

Findings in Parkinson disease, another neurodegenerative condition, further support the clinical relevance of separating the influences of thickness and surface area on volume loss. When re‐analyzing a volume‐based analysis to consider the differential impacts of thickness and surface area, Gerrits et al. (2016) found that these two measures correlated with different dimensions of cognitive performance in Parkinson disease patients. These findings suggest that separate and complete analysis of both thickness and surface area loss, rather than the combined measure of volume loss, offers a more meaningful view of cortical change than thickness, surface area, or volume loss alone.

While surface area loss represents a major component of the volume loss seen in AD (Figure 2), change in surface area has not been as widely studied as change in thickness or change in volume. This omission may reflect the technical limitations of commonly used methods, particularly when evaluating small cohorts (Figures 6 and 7). Notably, at the small sample sizes used in this study, the FreeSurfer longitudinal pipeline was entirely unable to detect significant differences in surface area loss between diagnostic groups. ROI analysis was able to detect significant differences in certain predefined regions, such as the middle temporal gyrus, but this methodology sacrifices detail by forcing results to conform to predefined ROIs. Improvements in the vertex‐wise measurement of change in surface area, such as those possible with aMSM, provide a critical tool in disentangling the influences of thickness and surface area loss on cortical atrophy in AD and other neurodegenerative diseases.

### 4.2 Surface area loss as a more precise measure of cortical atrophy

While thickness and volume loss remain important measures of atrophy, our results suggest that change in surface area, when precisely and accurately mapped using a tool such as aMSM, possesses considerable utility as an independent measure of atrophy. To illustrate the performance of each metric, we first considered a relatively small subset of subjects from the ADNI1 dataset. When we compared maps of atrophy derived from the same subjects over the same period of time, but scanned at different field strengths, we found that change in surface area was more consistent across field strengths than was change in thickness (Figure 8). This suggests that change in surface area may offer increased precision over change in thickness when analyzing scans of varying image quality. We also observed that, when comparing results obtained using a range of sample sizes, trends in surface area loss were more consistent across sample sizes than were trends in thickness loss (Figures 9 and 10). In this case, surface area loss offered increased statistical sensitivity over thickness loss when considering small samples. While volume loss, the combination of thickness and surface area loss, tended to be most sensitive across sample sizes (Figures 9, 10, and S6), it suffered from the loss of precision inherent in the thickness measure (Figure S5).

The decreased precision associated with measures of change in thickness is not surprising, given that the thickness of the cortex ranges from 1 to 4.5 mm (Fischl & Dale, 2000), and MRI scans, including those used in this study (Jack et al., 2008), are often acquired with a target isotropic voxel size of 1 mm^3^. In these conditions, even minimal bias or measurement error can become problematic when calculating local change. To compound this problem, thickness maps calculated using surface‐based methods may introduce bias (Scott, Bromiley, Thacker, Hutchinson, & Jackson, 2009), and precision is further compromised by the use of smoothing procedures (Bernal‐Rusiel et al., 2010). These issues become particularly problematic when attempting to measure change within a single subject. By contrast, the surface area attributed to any vertex considered in this analysis is on the order of 20 mm^2^, and high‐quality reconstruction of the cortical surface represents the original goal of popular reconstruction packages such as FreeSurfer.

This highlights a final benefit associated with maps of change in surface area generated by aMSM, which has been optimized to produce physically accurate surface deformations (Robinson et al., 2018). While additional work is needed to optimize the thickness dimension for atrophy mapping at the individual subject level, the results in this paper suggest that information gleaned from surface area alone is highly accurate and may be clinically useful to highlight unique regions undergoing cortical atrophy. This ability to track cortical changes in individual subjects offers a foundation to enable more personalized patient care and has the potential to aid in the implementation of precision medicine in AD detection and management.

### 4.3 Limitations

We note that, while surface area loss, measured with aMSM, offers advantages over thickness loss in small sample sizes, sample size remains an important consideration in study design. In this study, about 20% of potential subjects considered for our primary 3.0 T analysis were excluded due to failed or poor‐quality segmentation with FreeSurfer. This suggests that future analyses may benefit from consideration of alternate segmentation tools. Particularly in studies of Alzheimer's disease, tools such as MaCRUISE (Huo et al., 2016), which has demonstrated efficacy in older populations, may allow for a higher segmentation success rate. While the focus of this paper has been to establish the benefits of aMSM as a registration tool, studies of cortical atrophy may find additional advantage in the careful selection of a segmentation tool suited to the population of study.

We also acknowledge that multi‐scanner, multi‐site data can be complicated by unwanted scanner effects. While we have attempted to minimize these effects by using standardized datasets and balancing our diagnostic groups in terms of site and scanner manufacturer, we did not account for other differences in MRI protocol that may exist, which could confound physiologic trends. Furthermore, differences between 1.5 and 3.0 T results must be interpreted cautiously, since scanner type and manufacturer vary dramatically between these two datasets (Tables 2, 4, and S6–S9). Future studies using ADNI data or other multi‐site datasets may benefit from the implementation of a more comprehensive data harmonization process, such as the ComBat (Johnson, Li, & Rabinovic, 2007) derived methods employed in some recent analyses of structural MRI scans (Beer et al., 2020; Pomponio et al., 2020). In this study, longitudinal measures for each individual (surface area loss, thickness loss, and volume loss) were calculated from scans collected on the same scanner, which may mitigate some scanner effects. However, trends within and between diagnostic groups may be expected to increase in accuracy with improved data harmonization. As such, data harmonization remains an important consideration and a direction for future study which cannot be overlooked.

## 5 CONCLUSION

In summary, this study presents a viable method of accomplishing longitudinal registration and producing maps of cortical surface area loss. With this approach, we identified atrophy at similar levels of significance using either maps of change in thickness or change in surface area. Notably, however, surface area maps did not require smoothing and thus avoided a source of lost precision present in our thickness‐based analysis. We illustrated that aMSM is more effective than existing methods at detecting surface area loss, producing improved maps of surface area loss that can benefit future studies seeking to elucidate the precise contributions of thickness and surface area to atrophy in AD and other neurodegenerative disorders. These maps are also a promising means of evaluating small subpopulations, due to their increased precision compared to maps of change in thickness or change in volume. Finally, this method offers the potential for improved mapping of atrophy in individual subjects, an advancement that could prove clinically useful in AD detection and management.

## CONFLICT OF INTEREST

The authors declare no conflict of interest.

## Supporting information


**APPENDIX S1**: Supporting Information

## Data Availability

The data that support the findings of this study are available from the corresponding author upon reasonable request.
