# Supplementary material for: Enhanced detection of cortical atrophy in Alzheimer's disease using structural MRI with anatomically constrained longitudinal registration
Source: Hum Brain Mapp. 2021 May 14;42(11):3576–92. doi: 10.1002/hbm.25455 (PMC8249882; doi:10.1002/hbm.25455)
Supplement: Supplementary file 1 — APPENDIX S1: Supporting Information [file HBM-42-3576-s001.docx]

**Supplementary material**

**Enhanced detection of cortical atrophy in Alzheimer’s disease using structural MRI with anatomically-constrained longitudinal registration**

Emily Iannopollo^1^, Kara Garcia^1,2^, for the Alzheimer’s Disease Neuroimaging Initiative*

^1^Indiana University School of Medicine

^2^Indiana University School of Medicine, Department of Radiology & Imaging Sciences

*Data used in preparation of this article were obtained from the Alzheimer’s Disease Neuroimaging Initiative (ADNI) database (adni.loni.usc.edu). As such, the investigators within the ADNI contributed to the design and implementation of ADNI and/or provided data but did not participate in analysis or writing of this report. A complete listing of ADNI investigators can be found at: http://adni.loni.usc.edu/wp-content/uploads/how_to_apply/ADNI_Acknowledgement_List.pdf

**Table S1**: ADNI1 3.0T cohort assessment sites

| Site ID | CN | MCI-S | MCI-C | AD |
| --- | --- | --- | --- | --- |
| 1 | 0 | 1 | 0 | 1 |
| 2 | 2 | 0 | 0 | 0 |
| 4 | 1 | 0 | 0 | 0 |
| 5 | 1 | 0 | 1 | 1 |
| 11 | 3 | 2 | 5 | 1 |
| 13 | 0 | 0 | 1 | 4 |
| 15 | 0 | 1 | 1 | 1 |
| 16 | 1 | 0 | 0 | 0 |
| 18 | 0 | 1 | 0 | 0 |
| 32 | 0 | 1 | 0 | 0 |
| 56 | 0 | 0 | 1 | 0 |
| 58 | 0 | 0 | 1 | 0 |
| 101 | 2 | 1 | 2 | 1 |
| 108 | 1 | 0 | 0 | 1 |
| 111 | 0 | 1 | 1 | 0 |
| 113 | 0 | 0 | 0 | 1 |
| 117 | 1 | 2 | 0 | 1 |
| 123 | 0 | 2 | 0 | 1 |
| Total | 12 | 12 | 13 | 13 |

**Table S2**: ADNI1 1.5T cohort assessment sites

| Site ID | CN | MCI-S | MCI-C | AD |
| --- | --- | --- | --- | --- |
| 1 | 2 | 2 | 2 | 2 |
| 2 | 1 | 2 | 6 | 1 |
| 3 | 0 | 0 | 1 | 2 |
| 4 | 0 | 0 | 0 | 1 |
| 5 | 1 | 1 | 4 | 3 |
| 6 | 3 | 0 | 1 | 1 |
| 8 | 4 | 0 | 0 | 0 |
| 9 | 2 | 4 | 2 | 3 |
| 10 | 3 | 4 | 2 | 2 |
| 11 | 4 | 3 | 7 | 5 |
| 12 | 1 | 0 | 0 | 1 |
| 13 | 2 | 3 | 4 | 6 |
| 14 | 3 | 5 | 1 | 1 |
| 15 | 0 | 0 | 2 | 1 |
| 16 | 0 | 0 | 3 | 1 |
| 17 | 4 | 4 | 3 | 3 |
| 18 | 3 | 4 | 1 | 0 |
| 19 | 3 | 0 | 0 | 0 |
| 20 | 2 | 1 | 1 | 1 |
| 30 | 1 | 1 | 1 | 4 |
| 32 | 1 | 1 | 2 | 2 |
| 33 | 1 | 1 | 0 | 1 |
| 36 | 0 | 2 | 1 | 1 |
| 39 | 0 | 0 | 1 | 0 |
| 41 | 5 | 2 | 1 | 0 |
| 44 | 2 | 1 | 2 | 1 |
| 45 | 2 | 3 | 1 | 3 |
| 52 | 5 | 4 | 6 | 6 |
| 56 | 0 | 0 | 0 | 1 |
| 57 | 2 | 3 | 2 | 0 |
| 58 | 0 | 2 | 1 | 0 |
| 62 | 2 | 2 | 4 | 3 |
| 86 | 1 | 2 | 0 | 1 |
| 87 | 2 | 3 | 0 | 2 |
| 94 | 2 | 1 | 3 | 3 |
| 101 | 3 | 1 | 2 | 3 |
| 102 | 1 | 3 | 1 | 0 |
| 103 | 2 | 0 | 2 | 1 |
| 106 | 1 | 0 | 1 | 2 |
| 107 | 2 | 2 | 4 | 3 |
| 108 | 0 | 1 | 0 | 2 |
| 109 | 1 | 1 | 1 | 0 |
| 110 | 3 | 1 | 1 | 1 |
| 111 | 0 | 1 | 3 | 1 |
| 113 | 1 | 2 | 1 | 3 |
| 114 | 1 | 2 | 1 | 3 |
| 116 | 1 | 1 | 0 | 0 |
| 117 | 1 | 3 | 1 | 0 |
| 118 | 1 | 1 | 1 | 2 |
| 120 | 1 | 3 | 2 | 1 |
| 123 | 3 | 3 | 2 | 3 |
| 124 | 3 | 4 | 1 | 3 |
| 127 | 1 | 0 | 1 | 0 |
| Total | 90 | 90 | 90 | 90 |

**Table S3**: Subject demographics by diagnostic group in ADNI1 1.5T 12-subject groups

| Characteristic | CN | MCI-S | MCI-C | AD |
| --- | --- | --- | --- | --- |
| Total Subjects | 12 | 12 | 12 | 12 |
| Gender |  |  |  |  |
| Males | 6 | 9 | 7 | 9 |
| Females | 6 | 3 | 5 | 3 |
| Ethnicity |  |  |  |  |
| Hispanic or Latino | 0 | 0 | 1 | 0 |
| Not Hispanic or Latino | 12 | 12 | 11 | 12 |
| Race |  |  |  |  |
| Black or African American | 2 | 2 | 0 | 0 |
| White | 10 | 10 | 12 | 12 |
| Mean age (SD) at baseline (years) | 76.19 (6.04) | 74.99 (4.31) | 71.61 (8.51) | 76.71 (10.42) |
| Mean (SD) years education | 15.00 (3.05) | 15.75 (2.26) | 15.92 (3.75) | 15.33 (3.75) |

**Table S4**: Subject demographics by diagnostic group in ADNI1 1.5T 24-subject groups

| Characteristic | CN | MCI-S | MCI-C | AD |
| --- | --- | --- | --- | --- |
| Total Subjects | 24 | 24 | 24 | 24 |
| Gender |  |  |  |  |
| Males | 14 | 17 | 17 | 14 |
| Females | 10 | 7 | 7 | 10 |
| Ethnicity |  |  |  |  |
| Hispanic or Latino | 0 | 1 | 0 | 0 |
| Not Hispanic or Latino | 24 | 23 | 24 | 24 |
| Race |  |  |  |  |
| Asian | 0 | 0 | 1 | 0 |
| Black or African American | 0 | 1 | 0 | 0 |
| White | 24 | 23 | 23 | 24 |
| Mean age (SD) at baseline (years) | 76.12 (6.79) | 75.99 (5.89) | 77.82 (7.01) | 76.12 (6.79) |
| Mean (SD) years education | 15.21 (2.65) | 14.67 (2.71) | 15.83 (3.02) | 15.21 (2.65) |

**Table S5**: Subject demographics by diagnostic group in ADNI1 1.5T 48-subject groups

| Characteristic | CN | MCI-S | MCI-C | AD |
| --- | --- | --- | --- | --- |
| Total Subjects | 48 | 48 | 48 | 48 |
| Gender |  |  |  |  |
| Males | 25 | 27 | 35 | 27 |
| Females | 23 | 21 | 13 | 21 |
| Ethnicity |  |  |  |  |
| Hispanic or Latino | 2 | 2 | 1 | 0 |
| Not Hispanic or Latino | 46 | 46 | 47 | 47 |
| Unknown | 0 | 0 | 0 | 1 |
| Race |  |  |  |  |
| Asian | 1 | 1 | 3 | 0 |
| Black or African American | 2 | 0 | 2 | 2 |
| White | 45 | 47 | 43 | 46 |
| Mean age (SD) at baseline (years) | 76.32 (5.63) | 74.63 (6.04) | 75.17 (7.41) | 74.27 (7.82) |
| Mean (SD) years education | 15.44(2.53) | 14.94 (2.58) | 16.02 (2.51) | 14.73 (3.25) |

**Table S6**: MRI scanner manufacturers for ADNI1 1.5T 12-subject groups

| Manufacturer | CN | MCI-S | MCI-C | AD |
| --- | --- | --- | --- | --- |
| GE Medical Systems | 5 | 6 | 4 | 7 |
| Philips Medical Systems | 1 | 0 | 1 | 1 |
| Siemens | 6 | 6 | 7 | 4 |
| Total | 12 | 12 | 12 | 12 |

**Table S7**: MRI scanner manufacturers for ADNI1 1.5T 24-subject groups

| Manufacturer | CN | MCI-S | MCI-C | AD |
| --- | --- | --- | --- | --- |
| GE Medical Systems | 11 | 15 | 11 | 10 |
| Philips Medical Systems | 2 | 0 | 2 | 1 |
| Siemens | 11 | 9 | 11 | 13 |
| Total | 24 | 24 | 24 | 24 |

**Table S8**: MRI scanner manufacturers for ADNI1 1.5T 48-subject groups

| Manufacturer | CN | MCI-S | MCI-C | AD |
| --- | --- | --- | --- | --- |
| GE Medical Systems | 25 | 30 | 27 | 25 |
| Philips Medical Systems | 4 | 3 | 1 | 7 |
| Siemens | 19 | 15 | 20 | 16 |
| Total | 48 | 48 | 48 | 48 |

**Table S9**: MRI scanner manufacturers for subjects with both 1.5T and 3.0T scans

| Manufacturer | CN | MCI-S | MCI-C | AD |
| --- | --- | --- | --- | --- |
| GE Medical Systems |  |  |  |  |
| 1.5T | 3 | 6 | 6 | 10 |
| 3.0T | 2 | 1 | 0 | 2 |
| Philips Medical Systems |  |  |  |  |
| 1.5T | 4 | 0 | 0 | 1 |
| 3.0T | 4 | 4 | 3 | 3 |
| Siemens |  |  |  |  |
| 1.5T | 4 | 3 | 6 | 1 |
| 3.0T | 5 | 4 | 9 | 7 |
| Total |  |  |  |  |
| 1.5T | 11 | 9 | 12 | 12 |
| 3.0T | 11 | 9 | 12 | 12 |


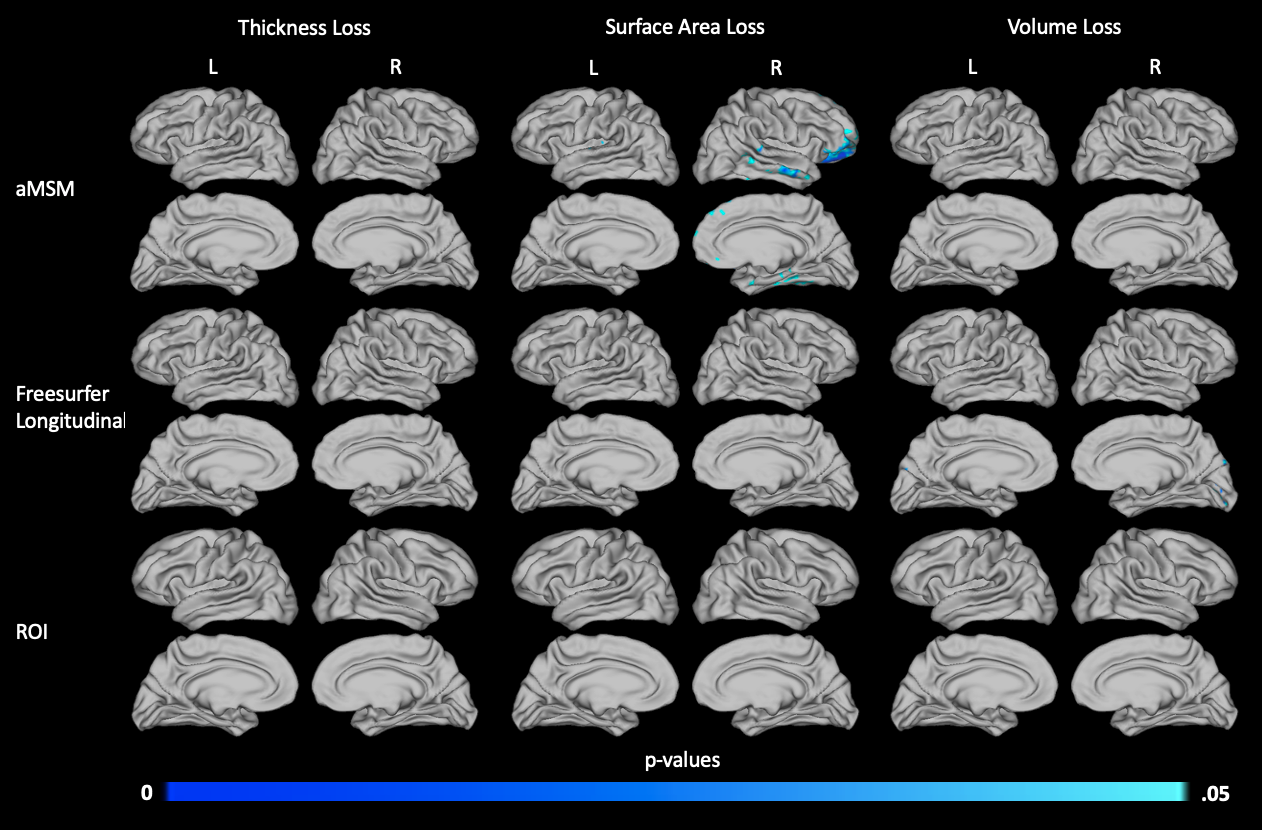


**Fig. S1:** Areas of significant thickness, surface area, and volume loss in the CN group (n=12), calculated using aMSM, the Freesurfer longitudinal pipeline, and predefined ROIs. P-values for the continuous surface maps produced using aMSM (top row) and the Freesurfer longitudinal pipeline (middle row) are threshold-free cluster enhanced with family-wise error correction. P-values associated with specific ROIs (bottom row) were adjusted with Bonferroni correction.


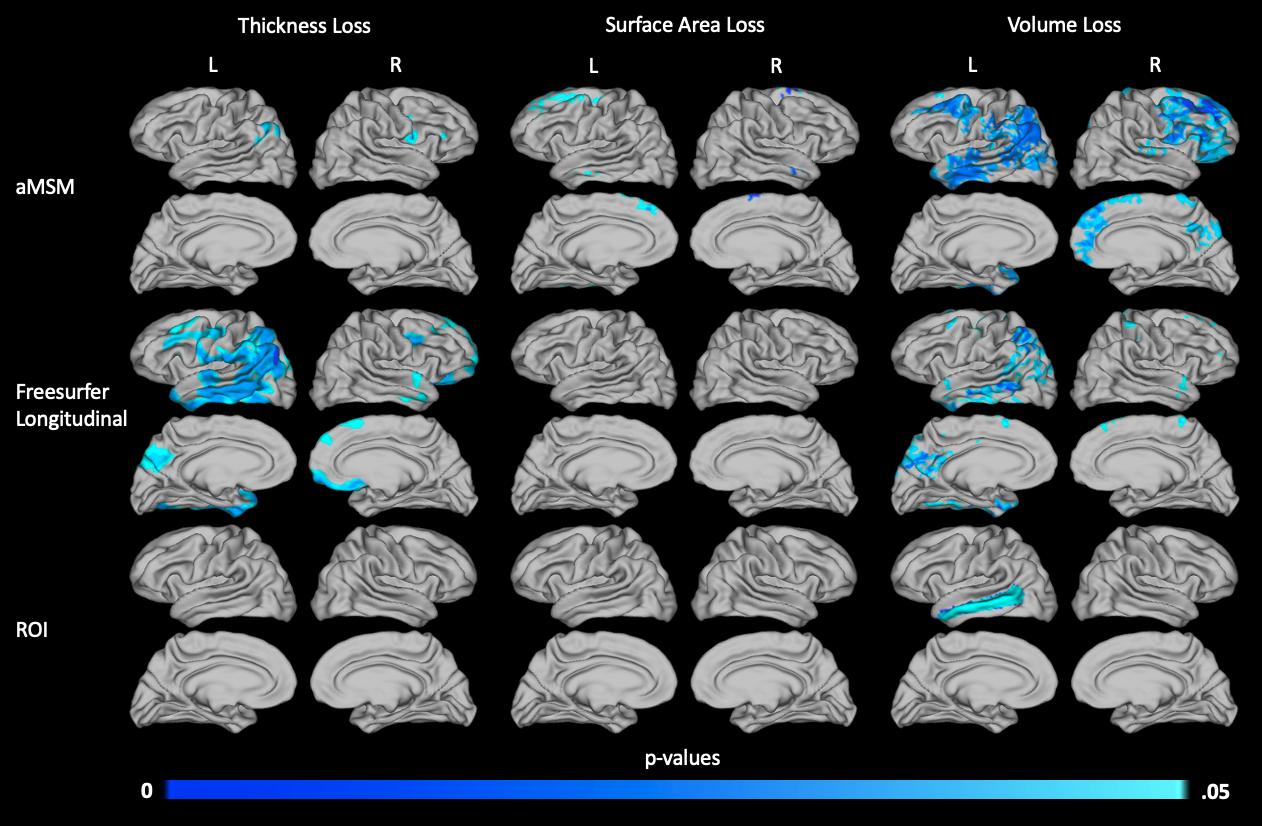


**Fig. S2:** Areas of significant thickness, surface area, and volume loss in the MCI-S group (n=12), calculated using aMSM, the Freesurfer longitudinal pipeline, and predefined ROIs. P-values for the continuous surface maps produced using aMSM (top row) and the Freesurfer longitudinal pipeline (middle row) are threshold-free cluster enhanced with family-wise error correction. P-values associated with specific ROIs (bottom row) were adjusted with Bonferroni correction.


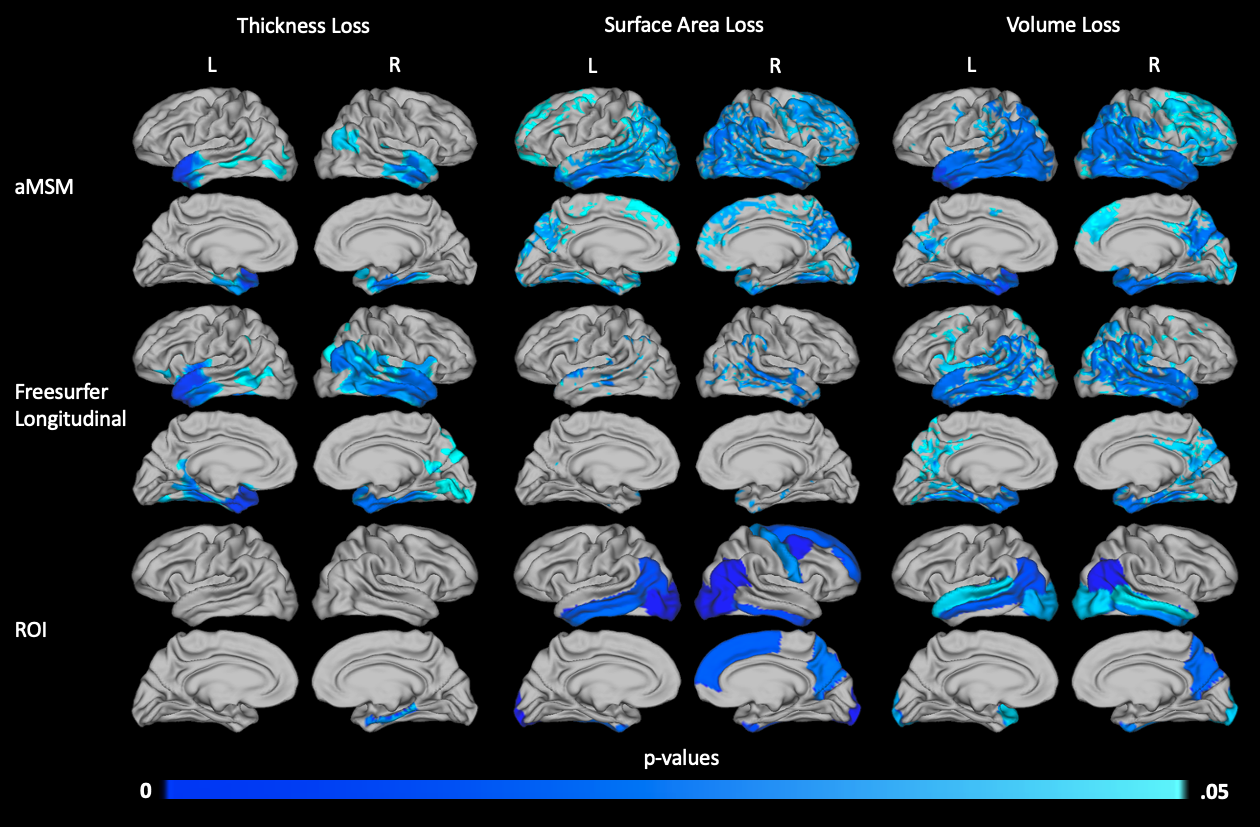


**Fig. S3:** Areas of significant thickness, surface area, and volume loss in the MCI-C group (n=13), calculated using aMSM, the Freesurfer longitudinal pipeline, and predefined ROIs. P-values for the continuous surface maps produced using aMSM (top row) and the Freesurfer longitudinal pipeline (middle row) are threshold-free cluster enhanced with family-wise error correction. P-values associated with specific ROIs (bottom row) were adjusted with Bonferroni correction.


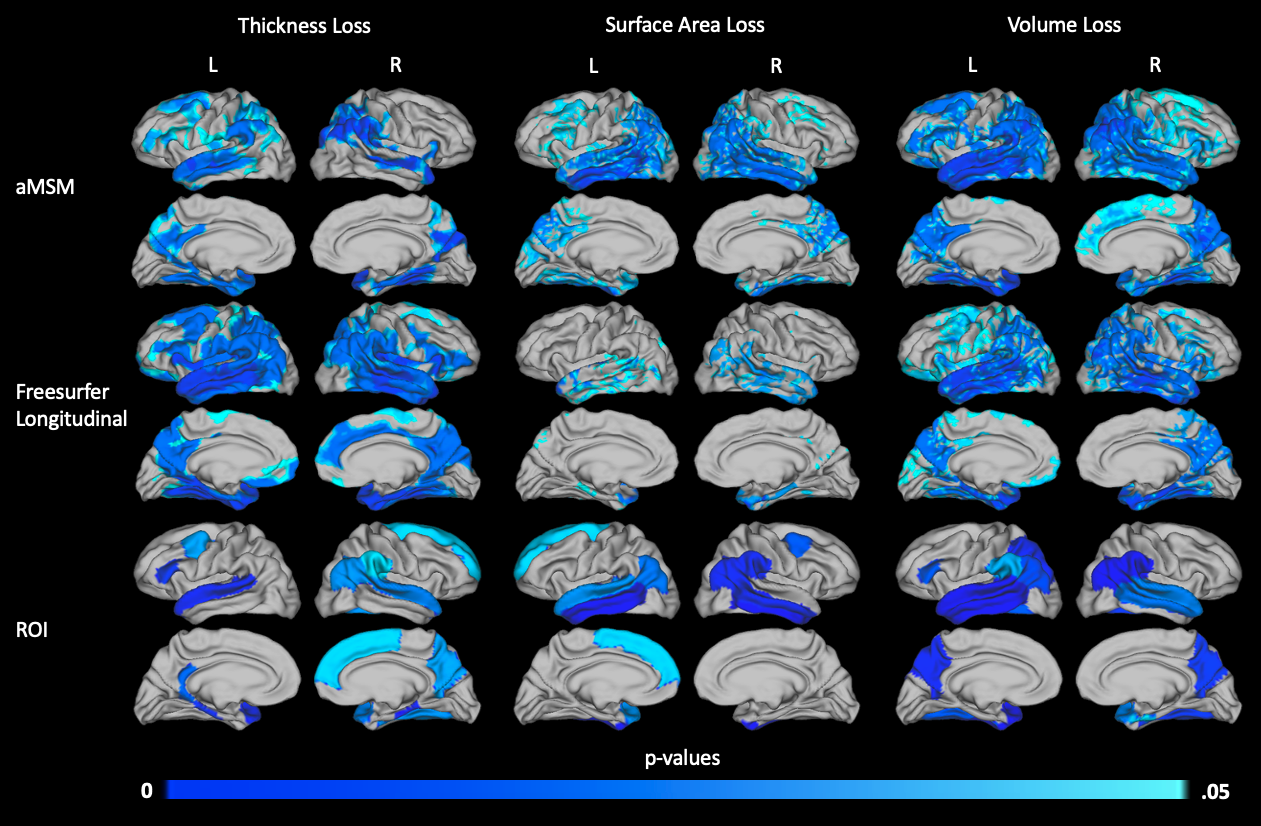


**Fig. S4:** Areas of significant thickness, surface area, and volume loss in the AD group (n=13), calculated using aMSM, the Freesurfer longitudinal pipeline, and predefined ROIs. P-values for the continuous surface maps produced using aMSM (top row) and the Freesurfer longitudinal pipeline (middle row) are threshold-free cluster enhanced with family-wise error correction. P-values associated with specific ROIs (bottom row) were adjusted with Bonferroni correction.


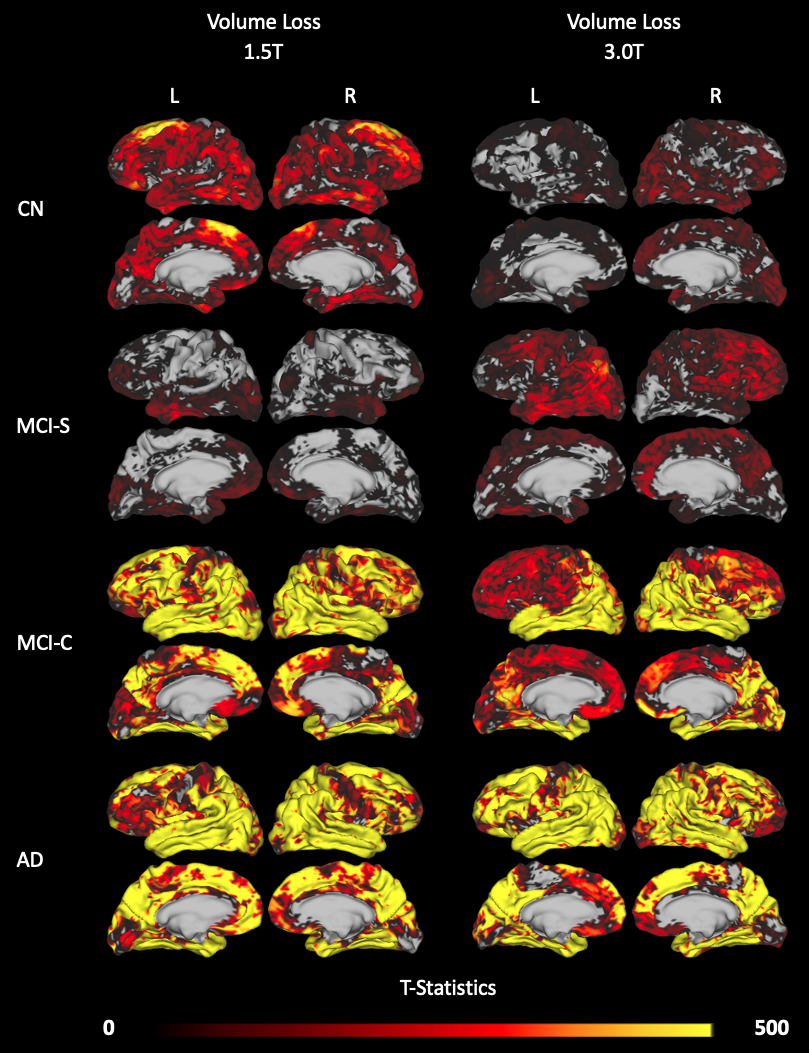


**Fig. S5:** Areas of volume loss over a two-year period assessed with 1.5T and 3.0T MRI.  Patterns of atrophy are shown for CN (n=11, top row), MCI-S (n=9, second row), MCI-C (n=12, third row), and AD (n=12, bottom row) groups.  Volume loss is shown as calculated using the 1.5T MRI scans (left column) and 3.0T scans (right column) of the same set of subjects over the same two-year period.  T-statistics are threshold-free cluster enhanced.


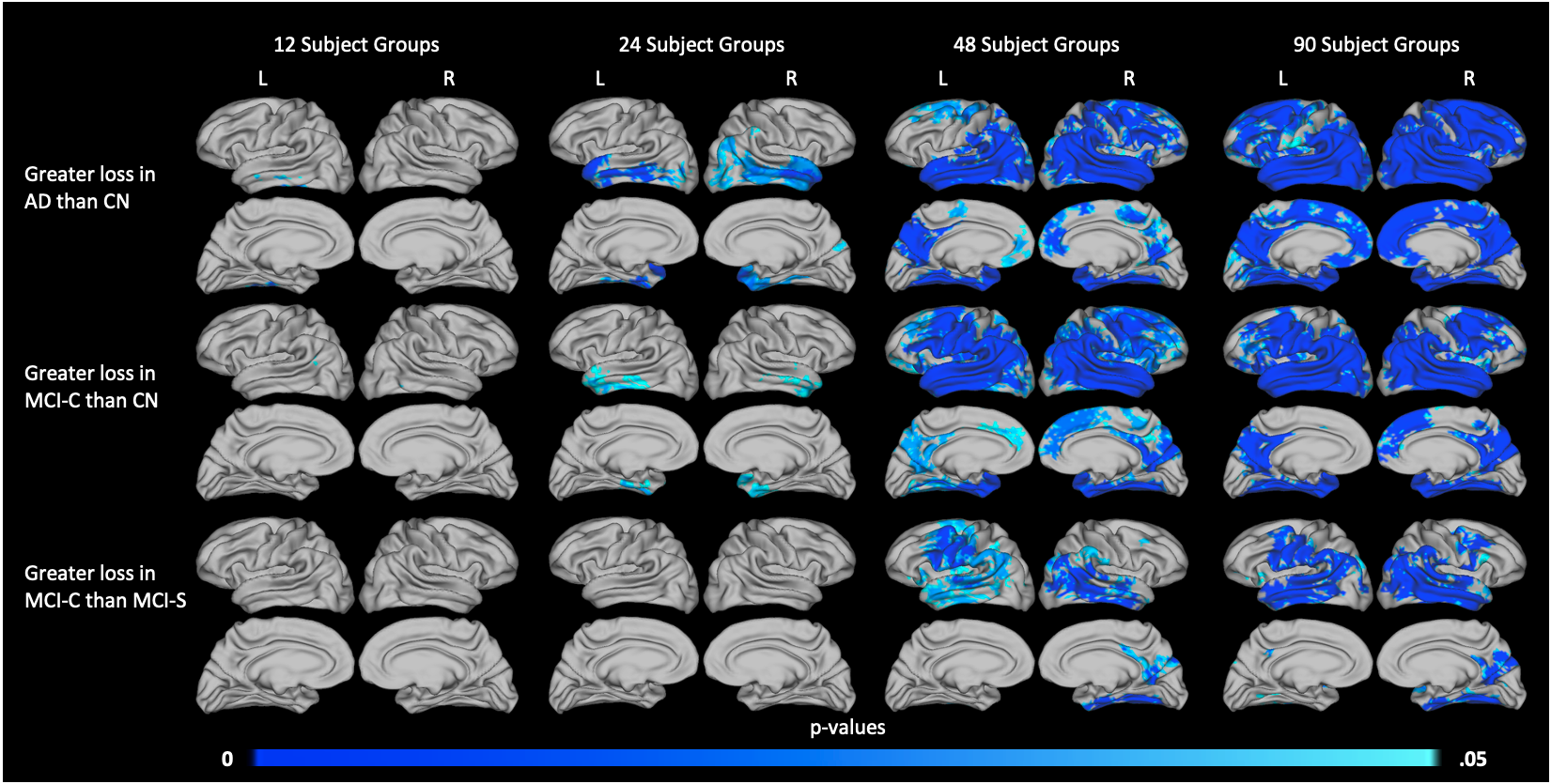


**Fig. S6**: Influence of sample size on the detection of volume loss. Significant differences in two-year atrophy between the AD and CN groups (top row), MCI-C and CN groups (middle row), and MCI-C and MCI-S groups (bottom row) are displayed. Results are shown for 12 subjects per diagnostic group (column 1), 24 subjects per diagnostic group (column 2), 48 subjects per diagnostic group (column 3), and 90 subjects per diagnostic group (column 4). All subjects were scanned at 1.5T. P-values are threshold-free cluster-enhanced with family-wise error correction.


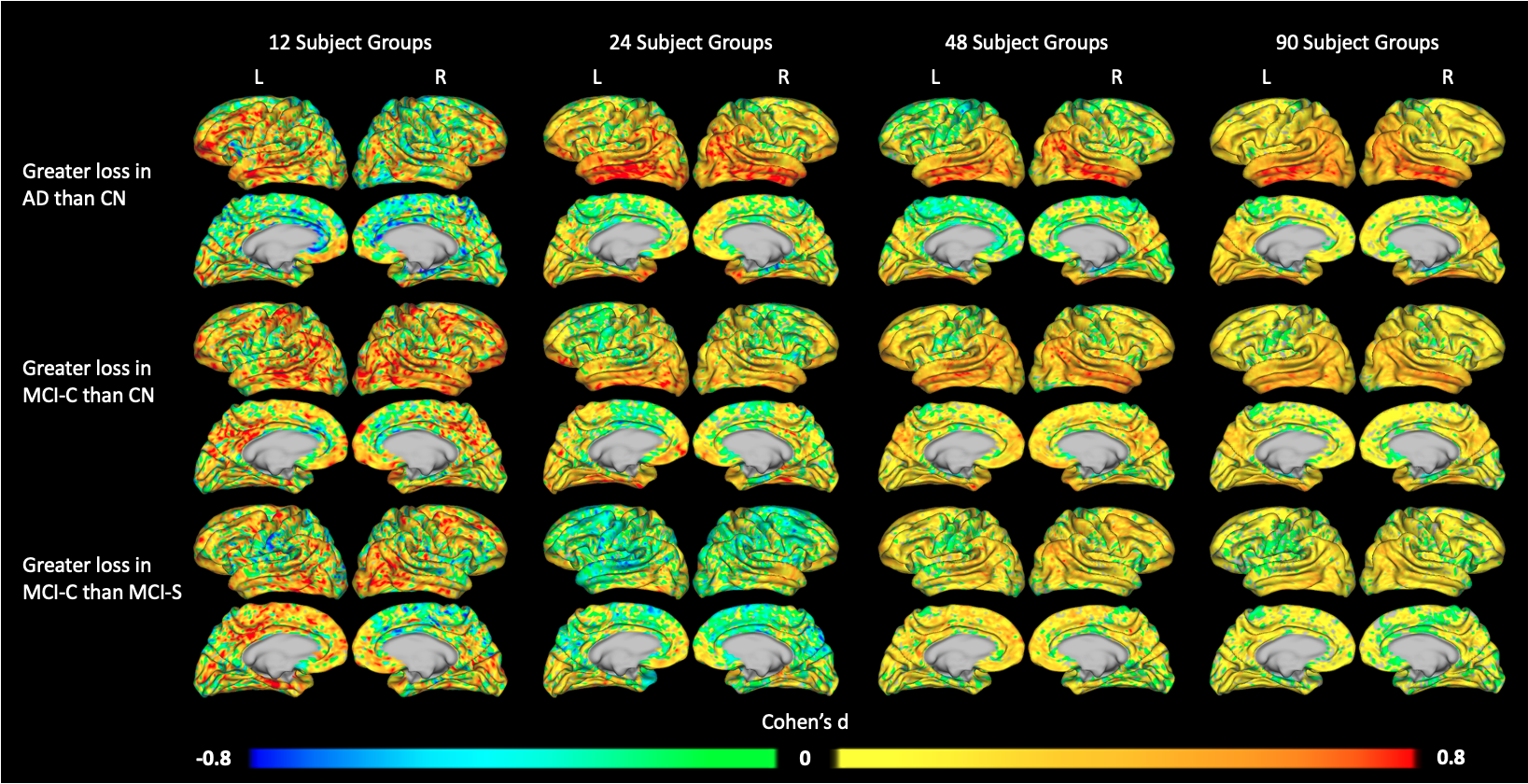


**Fig. S7**: Effect sizes associated with surface area loss, measured in groups of 12, 24, 48, and 90 subjects. Differences between the AD and CN groups (top row), MCI-C and CN groups (middle row), and MCI-C and MCI-S groups (bottom row) are displayed. Results are shown for 12 subjects per diagnostic group (column 1), 24 subjects per diagnostic group (column 2), 48 subjects per diagnostic group (column 3), and 90 subjects per diagnostic group (column 4). All subjects were scanned at 1.5T. Positive values for Cohen’s d indicate areas in which surface area decreased more in the AD (row 1) or MCI-C (rows 2, 3) group over the two-year period assessed. Negative values indicate areas in which surface area decreased more in the CN (rows 1, 2) or MCI-S (row 3) group over the two-year period assessed.


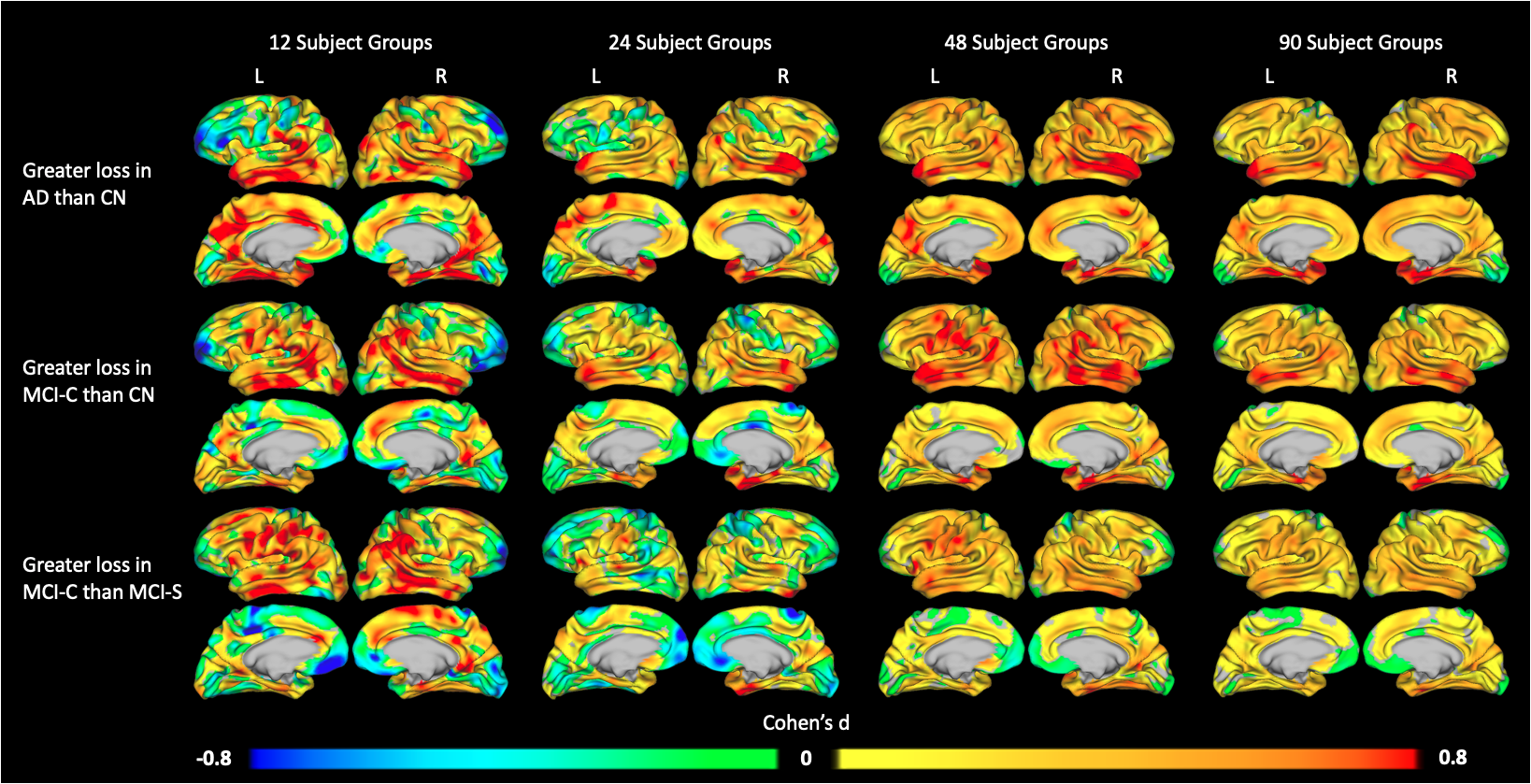


**Fig. S8**: Effect sizes associated with thickness loss, measured in groups of 12, 24, 48, and 90 subjects. Differences between the AD and CN groups (top row), MCI-C and CN groups (middle row), and MCI-C and MCI-S groups (bottom row) are displayed. Results are shown for 12 subjects per diagnostic group (column 1), 24 subjects per diagnostic group (column 2), 48 subjects per diagnostic group (column 3), and 90 subjects per diagnostic group (column 4). All subjects were scanned at 1.5T. Positive values for Cohen’s d indicate areas in which thickness decreased more in the AD (row 1) or MCI-C (rows 2, 3) group over the two-year period assessed. Negative values indicate areas in which thickness decreased more in the CN (rows 1, 2) or MCI-S (row 3) group over the two-year period assessed.
